# Vitiligo blood transcriptomics provides new insights into disease mechanisms and identifies potential novel therapeutic targets

**DOI:** 10.1186/s12864-017-3510-3

**Published:** 2017-01-28

**Authors:** Rama Dey-Rao, Animesh A. Sinha

**Affiliations:** Department of Dermatology, Jacobs School of Medicine and Biomedical Sciences, University at Buffalo, 6078 Clinical and Translational Research Center, 875 Ellicott Street, Buffalo, NY 14203 USA

**Keywords:** Vitiligo, Microarray, Interactome, Autoimmune, Type I and II Interferon, Peripheral blood

## Abstract

**Background:**

Significant gaps remain regarding the pathomechanisms underlying the autoimmune response in vitiligo (VL), where the loss of self-tolerance leads to the targeted killing of melanocytes. Specifically, there is incomplete information regarding alterations in the systemic environment that are relevant to the disease state.

**Methods:**

We undertook a genome-wide profiling approach to examine gene expression in the peripheral blood of VL patients and healthy controls in the context of our previously published VL-skin gene expression profile. We used several *in silico* bioinformatics-based analyses to provide new insights into disease mechanisms and suggest novel targets for future therapy.

**Results:**

Unsupervised clustering methods of the VL-blood dataset demonstrate a “disease-state”-specific set of co-expressed genes. Ontology enrichment analysis of 99 differentially expressed genes (DEGs) uncovers a down-regulated immune/inflammatory response, B-Cell antigen receptor (BCR) pathways, apoptosis and catabolic processes in VL-blood. There is evidence for both type I and II interferon (IFN) playing a role in VL pathogenesis. We used interactome analysis to identify several key blood associated transcriptional factors (TFs) from within (*STAT1, STAT6* and *NF-kB*), as well as “hidden” (*CREB1, MYC, IRF4, IRF1,* and *TP53*) from the dataset that potentially affect disease pathogenesis. The TFs overlap with our reported lesional-skin transcriptional circuitry, underscoring their potential importance to the disease. We also identify a shared VL-blood and -skin transcriptional “hot spot” that maps to chromosome 6, and includes three VL-blood dysregulated genes (*PSMB8, PSMB9* and *TAP1*) described as potential VL-associated genetic susceptibility loci. Finally, we provide bioinformatics-based support for prioritizing dysregulated genes in VL-blood or skin as potential therapeutic targets.

**Conclusions:**

We examined the VL-blood transcriptome in context with our (previously published) VL-skin transcriptional profile to address a major gap in knowledge regarding the systemic changes underlying skin-specific manifestation of vitiligo. Several transcriptional “hot spots” observed in both environments offer prioritized targets for identifying disease risk genes. Finally, within the transcriptional framework of VL, we identify five novel molecules (*STAT1, PRKCD, PTPN6, MYC* and *FGFR2*) that lend themselves to being targeted by drugs for future potential VL-therapy.

**Electronic supplementary material:**

The online version of this article (doi:10.1186/s12864-017-3510-3) contains supplementary material, which is available to authorized users.

## Background

Vitiligo vulgaris (non-segmental vitiligo) is a socially debilitating depigmenting disorder with a prevalence of 0.5–1% in the world population without a pronounced gender bias [1–5]. VL is an acquired, chronic skin and hair condition characterized clinically by loss of skin pigment (melanin), which, if untreated, is typically progressive and irreversible.

The autoimmune hypothesis of VL has the widest support. Autoantibodies against membranous components of melanocytes were found to be present in patient sera [6–8], and have recently been investigated as predictors of disease progression [9]. Several autoimmune comorbidities including autoimmune thyroid disease, pernicious anemia, systemic lupus erythematosus (SLE), and Addison’s disease have also been reported in association with VL [10–14]. Moreover, 20% of patients have at least one first-degree relative with VL and other autoimmune disorders (AID) [15, 16], supporting the notion of shared genetic factors across various diseases. Similarities in pathogenetic mechanisms with alopecia areata suggest new treatment modalities [17]. However, the high degree of familial aggregation has a non-Mendelian pattern of inheritance which is indicative of VL being polygenic with the influence of a large number of factors in nature [18, 19]. Several putative susceptibility loci have been reported, [20–29] including human leukocyte antigen (HLA)-associated genes such as *HLA-A2, HLA-DR4* and *HLA-DR7* alleles. [20, 30, 31] Non-HLA immune regulators such as *ACE, CAT, CTLA4, ESR, MBL2, NALP1, FOXP1* and *IL2RA* [32, 33] and genes including *DDR1, XBP1*, *NLRP1*, *PTPN22,* and *COMT* have also been associated with VL [34].

The literature around VL suggests a generalized immune dysregulation that is at least in part genetically based, resulting in humoral [35] and/or cellular (T-cell) immune responses [36–40] directed at melanocytes. Both innate and adaptive immunity appear to play a role in disease progression [20]. Several antigenic proteins coded by genes such as *MLANA/MART1*, *PMEL* (melanosome related) as well as *TYR, TYRP1* and *TH* (tyrosine related) have been identified in vitiligo. There is evidence for a key role of cytotoxic T lymphocytes (CD8 + T cells) as well as cytokines including interferon-gamma (IFN-γ) in VL pathogenesis [40–51]. Nonetheless, despite a plethora of scientific literature, the full complement of genetic elements of susceptibility, and their role in disrupting immune (and non-immune) pathways remains to be clarified.

To advance the investigation of the genetic basis for disease, we examined differential gene expression in the peripheral blood of patients diagnosed with non-segmental VL as compared with healthy control individuals and placed this information in context of our previous gene expression analysis in VL skin. We integrated the transcriptional data with functional annotations, clinical criteria and knowledge of VL genetics in an *in silico* bioinformatics-based approach to develop a more comprehensive framework of disease through which novel molecules could be proposed for future targeted therapy. The *in silico* interactome analysis allowed us to define “over-connected” key transcriptional drivers of dysregulated pathways/processes in non-segmental vitiligo. We found a total of 12 VL-blood (6) and -skin (6) transcriptional “hot spots” offering several genes that can be prioritized targets for identifying disease risk genes in future. Finally, we carefully prioritize 5 molecular targets from VL-skin or blood (*STAT1, PRKCD, PTPN6, MYC* and *FGFR2*) that can potentially explored as VL therapies.

## Results

### Unbiased analysis separates samples based on “disease-state”

**Fig. 1:**
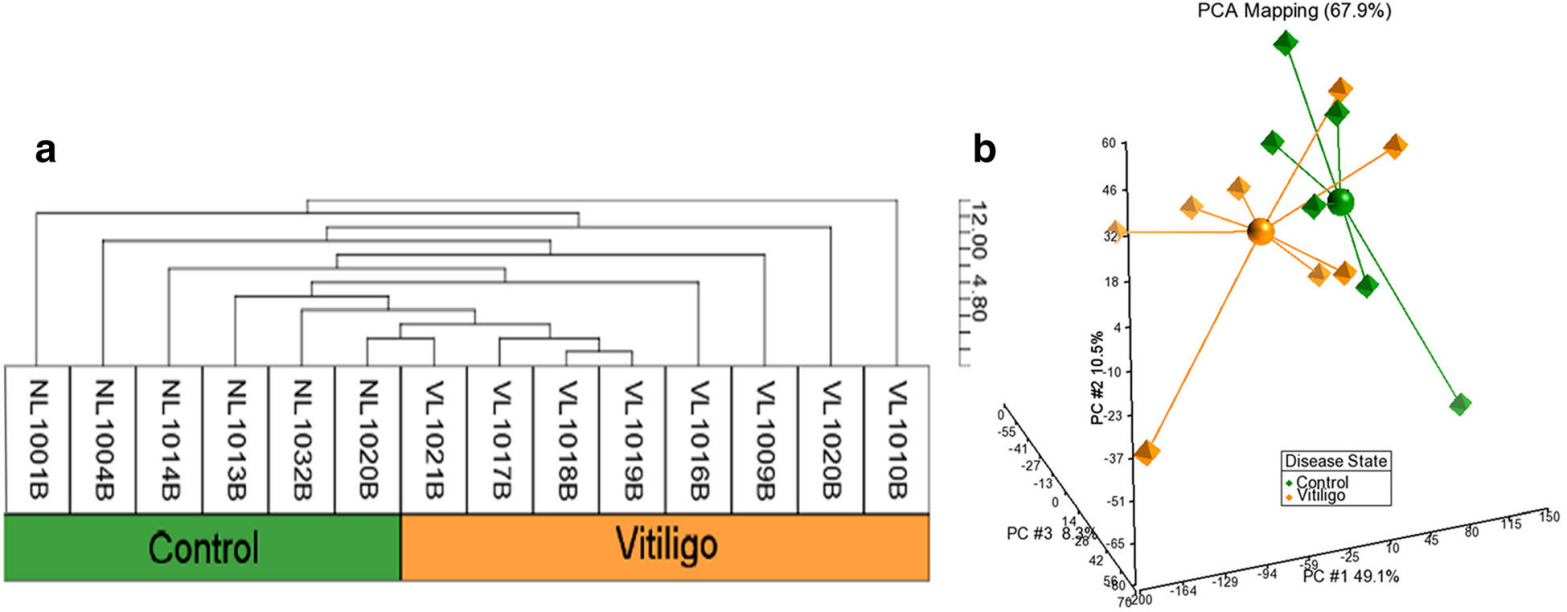
Unsupervised hierarchical clustering of VL-blood. Unsupervised hierarchical cluster analysis of the 1346 most variably expressed genes across the 14 arrays (blood samples of 8 VL patients and 6 healthy controls) was performed. **a** The dendrogram demonstrates a clustering of VL patients from healthy individuals revealing a molecular classification based on “disease-state”. **b** Principal components analysis (PCA) displays a scatter plot showing clear spatial separation of the samples (octahedrons) that are connected to a centroid sphere, with no outliers; In the 3-dimensional plot, the three principal components PC#1, #2 and #3 of all samples with all probeset IDs and their respective expression variations are plotted on the x-, y- and z- axes. The total percentage of PCA mapping variability is 67.9%. Color assignations of vitiligo patient (*orange*) and healthy controls (*green*) were made after examination of distribution of samples

Gene expression variations from peripheral blood is sufficient to segregate VL patients from healthy controls in a non-supervised hierarchical clustering analysis (Fig. 1a). Within this “disease-state” related signature, we subsequently report a group of 319 downregulated transcripts that is significantly enriched in Gene Ontology (GO) biological processes (BPs) correlated with regulation of immune response, cell activation, response to virus, leukocyte activation as well as inflammatory response, among others (Additional file 1: Table S1). Principal components analysis (PCA) showed a spatial separation of samples without outliers or batch effects (Fig. 1b). In summation, we were able to assign a blood gene expression signature capable of separating VL patients and healthy controls using unsupervised analytical methods.

### Pathways and processes based enrichment analyses of VL-DEGs

“Disease-state” was the largest source of variation capable of separating VL patients from healthy controls. Ninety-nine non-redundant differentially expressed genes (DEGs) (*p*-value <0.05, fold change (FC) cut off ≥ ±1.4) [4 up-regulated in patients (UIP) and 95 down-regulated in patients (DIP)] distinguished the two groups. The complete DEGs list is included in Additional file 2: Table S2. The top up- and down- regulated genes are included in Table 1a-b. Two members of the cytochrome P450 superfamily, *CYP3A5* (FC = 1.5) and *CYP1B1* (FC = 1.5), were among the top upregulated genes, and genes encoding IFN-induced proteins, *IFIT1* (FC = -4.0) *IF144L* (FC = -4.0), *IFIT3* (FC = -3.5) and *IFITM2* (FC = -2.7), were among the top down-regulated genes.

**Table 1:** (a) Top upregulated and (b) top down-regulated DEGs in vitiligo (VL)-blood expression profile

Entrez ID	Gene Symbol	Gene Title	*p*-value	Fold-Change
a				
2201	*FBN2*	fibrillin 2	0.0345	1.5
1577	*CYP3A5*	cytochrome P450, family 3, subfamily A, polypeptide 5	0.0315	1.5
23253	*ANKRD12*	ankyrin repeat domain 12	0.0236	1.4
1545	*CYP1B1*	cytochrome P450, family 1, subfamily B, polypeptide 1	0.0069	1.4
b				
9636	*ISG15*	ISG15 ubiquitin-like modifier	0.0172	−4.2
3434	*IFIT1*	interferon-induced protein with tetratricopeptide repeats 1	0.0168	−4.0
710	*SERPING1*	serpin peptidase inhibitor, clade G (C1 inhibitor), member 1	0.008	−4.0
10964	*IFI44L*	interferon-induced protein 44-like	0.0283	−3.6
4599	*MX1*	myxovirus (influenza virus) resistance 1, interferon-inducible protein p78 (mouse)	0.0098	−3.5
3437	*IFIT3*	interferon-induced protein with tetratricopeptide repeats 3	0.0133	−3.5
4277	*MICB*	MHC class I polypeptide-related sequence B	0.0085	−3.3
653361///654816///654817	*NCF1///NCF1B///NCF1C*	neutrophil cytosolic factor 1///neutrophil cytosolic factor 1B pseudogene///neutrophil	0.0436	−2.7
8638	*OASL*	2′-5′-oligoadenylate synthetase-like	0.0334	−2.7
10581	*IFITM2*	interferon induced transmembrane protein 2	0.0342	−2.7

Fold Change; a “positive” fold change indicates an upregulation and a “negative” fold change indicates a down-regulation in peripheral blood of VL patients vs. healthy controls

**Fig. 2:**
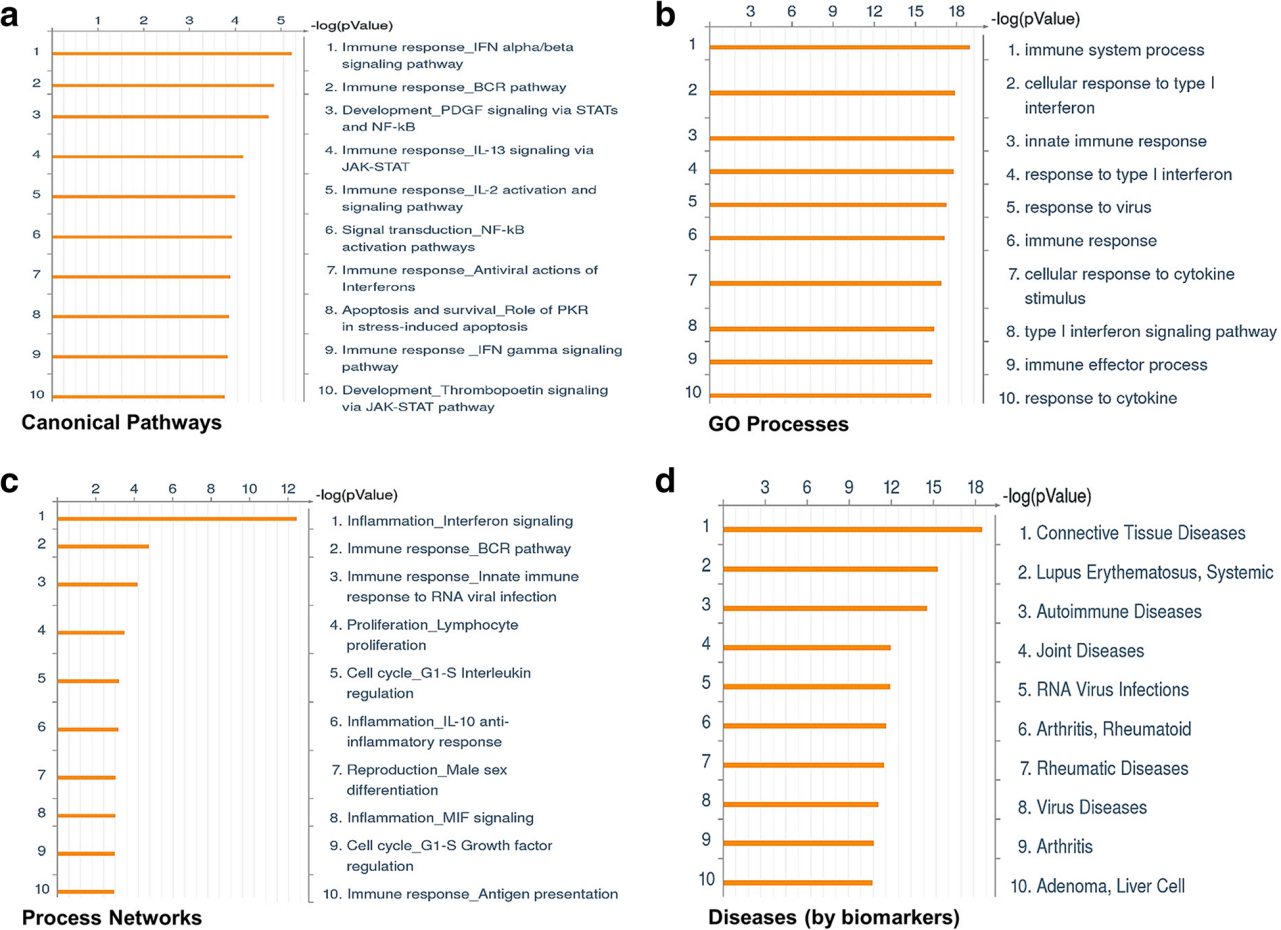
Functional annotation and pathway-based analysis of VL-blood associated transcriptional profile. Gene expression values from peripheral blood samples of 8 VL patients and 6 healthy individuals were compared, generating a trimmed list of 99 DEGs (4 up-and 91 down-regulated). Algorithms use the uploaded VL-blood associated DEGs as the input list and map each gene to a network object in the MetaCore database. Pathway and network analysis of the VL-blood transcriptional profile demonstrate an enrichment of the following categories: (**a**) canonical pathways (**b**) GO processes (**c**) process networks and (**d**) disease by biomarkers. Top ten enrichments are sorted and ranked by *p*-value shown on a logarithmic scale. A lower p-value indicates higher statistical significance

**Fig. 3:**
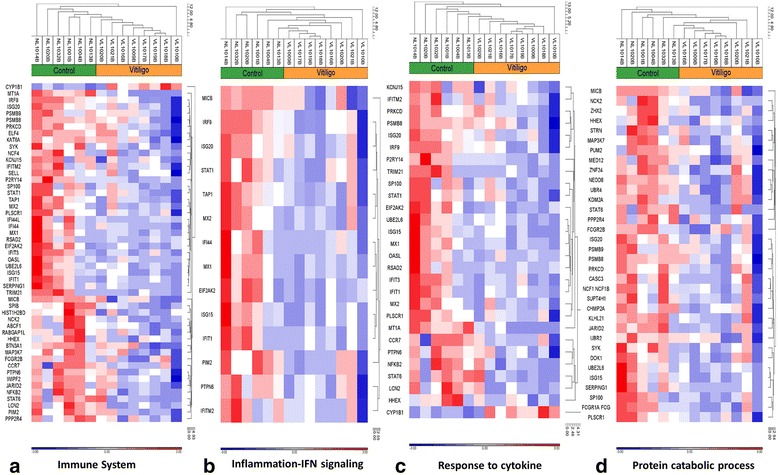
Disease-related GO pathways and processes enriched in VL-blood profile. Robust signals of: (**a**) immune system (51 DEGs), (**b**) IFN signaling and inflammation (14 DEGs), (**c**) response to cytokine (28 DEGs), and (**d**) protein catabolic process (34 DEGs), are revealed in the VL-blood DEGs. In the heat maps, red indicates upregulation while blue indicates down-regulation and white indicates unchanged expression. The blood samples cluster into VL patients (*orange*) and healthy controls (*green*). Expression value intensities are illustrated by the color of the scale with a range of -3.0 to +3.0 on a log scale

DEGs were processed through an "ontology enrichment analysis" via MetaCore (Fig. 2a-d) and DAVID (Additional file 3: Table S3 a, b) to identify prominent disease-related biological pathways/processes to better understand the pathobiology underlying vitiligo (Fig. 3a-d). Canonical pathways related to immune response including IFN α and β, BCR, interleukins, antiviral actions of IFNs, and NF-κB, and stress related apoptosis were among the most enriched in the VL-blood dataset (Fig. 2a, b). The significance of male sex-related process network (Fig. 2c) is not completely understood at this time since there is no reported sex-bias for VL [1]. Enriched diseases, such as those related to connective tissue (37 DEGs), SLE (30 DEGs) and autoimmune diseases (AID) (39 DEGs) (Fig. 2d) further support the relevance of our data based on reports of comorbidity in vitiligo patients [52–54].

**Fig. 4:**
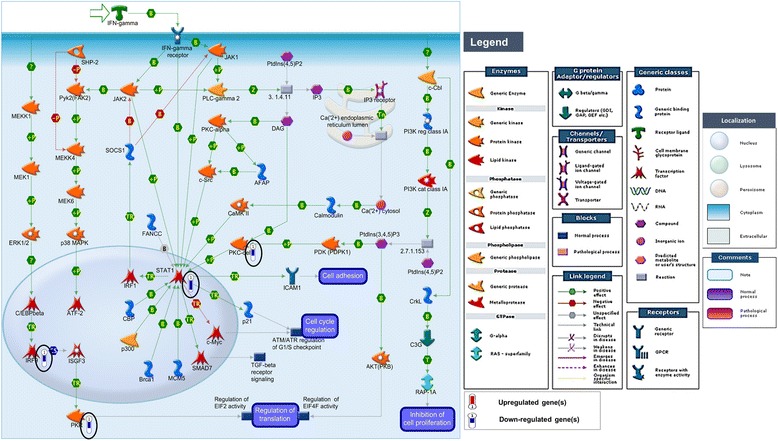
IFN-gamma signaling pathway. The IFN-γ signaling pathway is one of the top canonical pathways enriched (*p*-value 0.0001, FDR 0.005) in the VL-blood profile. Histograms of down-regulated genes from VL-blood (circled in *black*) are demonstrated in the map. The height of the histograms corresponds to the relative expression value for a particular gene/protein. Objects and interactions as well as direction of regulation are described in the legend

More than 50% of the VL-blood DEGs (51 DEGs; 50 DIP and 1 UIP) were associated with a prominent dysregulated immune system (Fig. 3a) which could be broadly divided into several categories: **a)** Type I IFN signaling (IFN α/β) [*IFIT1, IFITM2, IRF9, ISG15, ISG20, MX1 MX2, OASL, PTPN6, PSMB8,* (Additional file 4: Figure S1)]; **b)** Type II IFN (IFN-γ) signaling pathway [*IRF9, PRKCD, EIF2AK2 (PKR), STAT1* (Fig. 4)]; **c)** IFN signaling related to inflammation [*MICB, IRF9, ISG20, STAT1, TAP1, MX2, IFI44, PTPN6* (Fig. 3b)]; **d)** Cytokine related pathways (28 in total) (Fig. 3c) (IL-13 signaling (*STAT1, PTPN6, STAT6, PRKCD*] and IL-2 activation (*NFKB2, HMGA1, PTPN6* and *SYK*, among others). Expression of the 99 DEGs was tightly coordinated across all samples supporting their use as potential biomarkers of the “disease-state” in peripheral blood of patients (Additional file 5: Figure S2).

Several down-regulated genes in VL patients were involved in catabolic processes such as negative regulation of cellular protein, interferon stimulated gene-15 (ISG-15) protein conjugation, and proteolysis (Fig. 3d). Additionally, *FCGR2B, NCF1, FCGR1A, PRKCD,* and *SYK* form part of the Fc gamma R-mediated phagocytosis pathway, (Additional file 3: Table S3a) and *MICB, NFKB3, PSMB9, STAT1* and *TAP1* are associated with antigen presentation (Fig. 2c), which are all indicative of disrupted protein breakdown accompanied by faulty antigen processing and presentation.

### *In silico* analyses to investigate functionality of DEGs

VL-specific gene regulation was evaluated through an enrichment by “protein function” revealing a significantly high number (14) of transcription factors (TFs) and enzymes (18) (Additional file 6: Table S4). We prioritized potentially relevant individual genes, or hubs where essential genes are highly connected [55]. The topology uncovered a higher level of in-coming and out-going connections to and from the VL-dataset to the metabase with a concomitant higher clustering coefficient than either only among objects in the metabase or within the VL-blood experimental dataset (Additional file 7: Table S5a). Our analysis revealed 16 DEGs (*IRF9, STAT1, FCGR2B, PRKCD, PSMB9, PSMB8, PTPN6, MX1, MX2, IFIT1, TOMM34, IFIT3, SERPING1, NCF4, TAP1* and *ISG15)* in the VL-blood profile (representing TFs, receptors, kinases, proteases, phosphatases, enzymes and other general proteins) that are significantly “over-connected” with objects both within the VL-blood profile as well as the larger metabase (Table 2). *STAT1* and *IRF9* are the most significant TFs from the VL-blood experimental dataset (Additional file 7: Table S5b) that form a central hub of in-coming and out-going (positive and negative) interactions with the “over-connected” objects (Additional file 8: Figure S3). The network includes portions of several canonical pathways such as *IFN-α-IRF9-IFN-β, IFN-β-STAT1-MxA*, *IFN-γ-IFN-γ receptor-MxA*, major histocompatibility complex (MHC class I) interactions as well as *CASP8-cyt c-CASP9* tethering biological processes such as responses to immunity, defense, stress, signal transduction, apoptosis and regulation of proteolysis.

**Table 2:** Interactions by protein function. Sixteen "over-connected" genes in VL-blood expression profile

Protein Function	Gene IDs in active data set	Gene Title	Object name	Fold change	A	n	R	N	E	Ratio	z-score	*p*-value
TFs	IRF9	interferon regulatory factor 9	IRF9	−1.7	7	102	60	26494	0.231	30.3	14.1	3.32E-09
	STAT1	signal transducer and activator of transcription 1, 91kDa	STAT1	−2.6	22	102	575	26494	2.214	9.9	13.5	4.04E-16
Receptors	FCGR2B	Fc fragment of IgG, low affinity IIb, receptor (CD32)	Fc gamma RII beta	−1.4	2	102	35	26494	0.135	14.8	5.1	0.0080
Kinases	PRKCD	protein kinase C, delta	PKC-delta	−1.7	8	102	386	26494	1.486	5.4	5.4	0.0001
Proteases	PSMB9	proteasome (prosome, macropain) subunit, beta type, 9	PSMB9	−1.7	3	102	38	26494	0.146	20.5	7.5	0.0004
	PSMB8	proteasome (prosome, macropain) subunit, beta type, 8	PSMB8(LMP7)	−1.6	2	102	30	26494	0.115	17.3	5.6	0.0059
Phosphatases	PTPN6	protein tyrosine phosphatase, non-receptor type 6	SHP-1	−1.9	5	102	200	26494	0.770	6.5	4.8	0.0010
Enzymes	MX1; MX2	myxovirus (influenza virus) resistance 2 (mouse)	MxA	−3.5 ; -2.2	3	102	57	26494	0.219	13.7	6.0	0.0013
Others	IFIT1	interferon-induced protein with tetratricopeptide repeats 1	IFIT1	−4.0	4	102	15	26494	0.058	69.3	16.4	2.74E-07
	TOMM34	translocase of outer mitochondrial membrane 34	TOM34	−1.6	2	102	21	26494	0.081	24.7	6.8	0.0029
	IFIT3	interferon-induced protein with tetratricopeptide repeats 3	RIG-G	−3.5	6	102	66	26494	0.254	23.6	11.4	2.11E-07
	SERPING1	serpin peptidase inhibitor, clade G (C1 inhibitor), member 1	C1 inhibitor	−4.0	2	102	23	26494	0.089	22.6	6.4	0.0035
	NCF4	neutrophil cytosolic factor 4, 40kDa	p40-phox	−1.8	2	102	23	26494	0.089	22.6	6.4	0.0035
	TAP1	transporter 1, ATP-binding cassette, sub-family B (MDR/TAP)	TAP1 (PSF1)	−1.6	4	102	51	26494	0.196	20.4	8.6	4.5E-0
	ISG15	ISG15 ubiquitin-like modifier	ISG15	−4.2	8	102	256	26494	0.986	8.1	7.1	6.83E-06

Explanation of each column

Protein Function: overall associated functions with proteins; Gene IDs in active data sets: gene symbol associated with the VL skin transcriptional profile; Gene Title: Full name of gene; Object Name: network object in metabase; Fold change: expression by microarray VL vs control. A: number of network objects in the activated signatures which interact with the chosen object; n: number of network objects in the signature; R: number of network objects in the background list which interact with the chosen object; N: total number of protein-based objects in the background list; E (Expected): mean of hypergeometric distribution; Ratio: connectivity ratio (actual/expected); z-score: (actual-expected)/(standard deviation); p-value: probability to have the value of Actual or higher (lower for negative z-score) by chance under null hypothesis of no over- or under-connectivity; TFs: Transcription factors

“Over-connected” genes (encoded protein) by “function” is based on the connectivity of genes in the active dataset with genes from the Human Proteome in the MetaCore database (metabase). We found sixteen (MX1 and MX2 are both in the dataset and map to a group in metabase) significantly over-connected genes in VL-blood transcriptional profile where the number of observed interactions exceeded the number of expected interactions. There were no under-connected genes observed

**Fig. 5:**
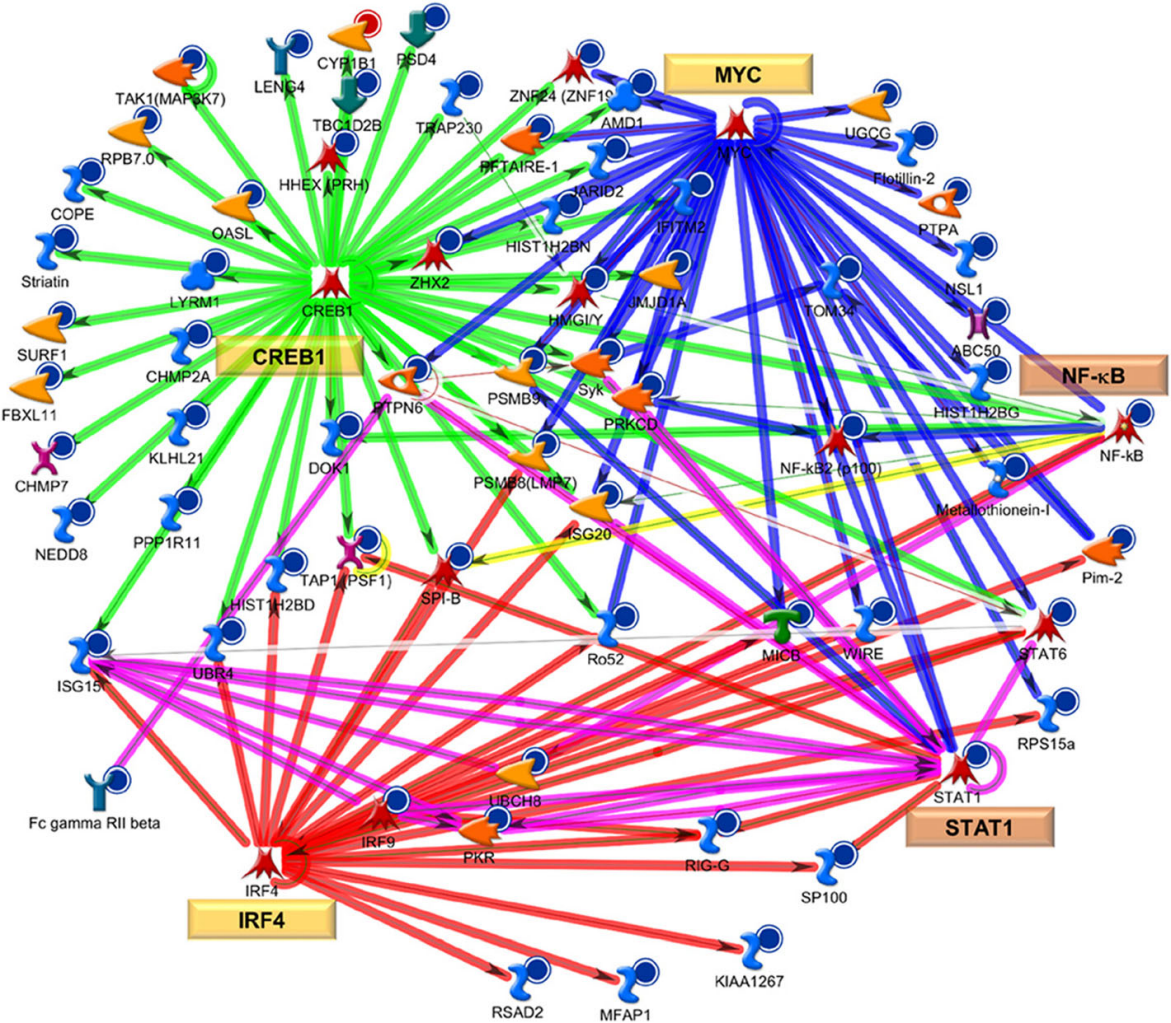
Network analysis of key transcriptional factors (TFs) in VL-blood pathology. The VL-blood DEGs list was analyzed using the “transcriptional regulation network” algorithm (see Additional file 9: Table S6). Auto-expanded (by one step) network was generated using the top three (*CREB1, MYC* and *IRF4*) TFs associated with disease-related BPs and then merged. *STAT1* and *NF-kB* (from the dataset- *orange* boxes) as well as *CREB1, MYC, IRF4,* (“hidden” from dataset- *yellow* boxes) are TF hubs revealing a large number of curated interactions directly to and from (direction of arrow) objects in the VL-blood profile. Each hub is color coded (red = IRF4, blue = *MYC*, green = *CREB1*, magenta = *STAT1/IRF9/ISG15* complex, variable colors = *NF-kB* (connected to several hubs). See Fig. 4 legend for types of objects

We investigated networks associated with TF encoding genes in the active dataset of potential importance to VL-blood pathology, and subjected the DEGs list to the “transcriptional regulation network” algorithm in MetaCore. Additional file 9: Table S6 summarizes 15 top scoring key TFs linked to networks tethering disease-related biological processes. Apart from significantly “over-connected” TF encoding genes such as *STAT1, IRF9* and *NF-kB* from within the VL-blood dataset, additional but “hidden” TFs such as *CREB1 MYC, IRF4, IRF1, SP1,* and *TP53* among others were revealed, echoing similar results obtained in our VL-skin analysis [56] where we had described *MYC* as a central TF in VL-pathology, as well as highlighted the relevance of significant “hidden” TFs. In summation, integrating our TF data from both VL-blood and skin (systemic and target tissue milieu) we find shared TF genes such as *MYC, CREB1, TP53, SP1, ESR1, and STAT1*, among others (which are either included/hidden in/from the VL-blood/skin dataset) that might be further evidence for their essential role in the transcriptional configuration underlying pathogenesis in VL. Figure 5 reveals TFs such as *CREB1*, *MYC*, *IRF4*, *STAT1* and *NF-kB* as hubs of in- and out- going interactions with several down-regulated genes in the VL-blood transcriptional profile.

### Searching for essential molecules for targeted VL-therapy

**Fig. 6:**
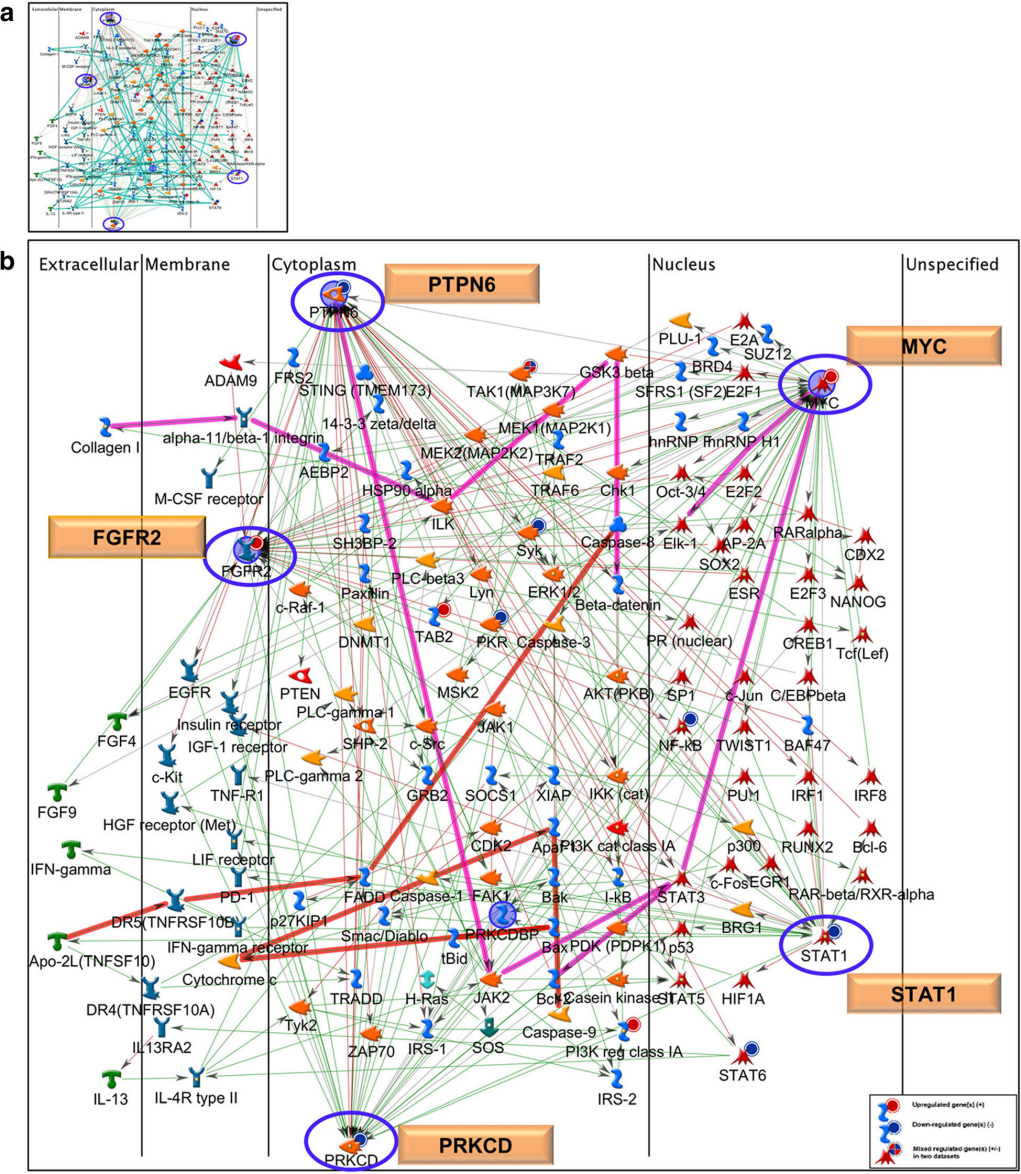
Network analysis using key molecules in VL pathogenesis. We prioritized five DEGs (*STAT1, MYC, PRKCD, PTPN6,* and *FGFR2*) based on combined transcriptional- (VL-blood and skin) as well as *in silico* bioinformatics-based analyses. These 5 molecules/proteins were used as seed nodes to construct a network using the shortest path algorithm with a 2-step connection. Proteins are ordered according to their cellular localization from left to right. All five prioritized molecules are reaction hubs (*blue* ovals) where curated interactions are observed with both positive (*green* lines) and negative effects (*red* lines) directly to and from (direction of arrow) the hubs with several objects in the VL-blood profile as well as the larger human proteome database. (**a**) Portions of several canonical pathways are included in the network (in *cyan*), (**b**) a few canonical pathway portions have been traced including *PTPN6-MYC-ELK1*, *Collagen 1-alpha11/beta1integrin-MYC*, *PTPN6-JAK2-BCL2* (in *magenta*) that are involved in immune regulation and inflammation and others such as *CASP8-Cytochrome c-CASP9*, *Apo2L-DR5-BAX* (in *orange*), are associated with apoptosis and survival. Gene expression data from both VL-blood and skin profiles are overlaid on the same network. The up- and down-stream interactions revealed by our network analyses may be leveraged to assess and validate functional consequences of targeting the 5 molecules by therapeutic drugs in future investigations. See Fig. 4 for legend

In a search for essential VL-related molecules, we selected 5 DEGs: *STAT1, PRKCD* and *PTPN6* from the blood as well as *MYC* and *FGFR2* from the skin transcriptional profiles. These molecules are strongly implicated as key players in the disease based upon the observations of: 1) dysregulated expression in VL skin or blood; 2) inclusion in disease-related pathways and processes; and 3) *in-silico* bioinformatics-based analyses highlighting functional significance. Network analysis starting with these five molecules (*STAT1, PRKCD*, *PTPN6, MYC* and *FGFR2)* as seed nodes, revealed them as reaction hubs with in-coming and out-going links (positive and negative influence) to several objects from the VL-skin and blood datasets as well as the larger metabase including portions of several canonical pathways (Fig. 6a). These pathways are associated with molecules such as IFN-γ, interleukins, growth and death receptors, caspases, kinases and transcriptional factors (Fig. 6b). Some of the VL-related GO biological processes tethered by these molecules are positive regulation of cellular metabolic process, response to stress, immune response, signal transduction, defense, cell proliferation as well as apoptosis, among others. Finally, the five key functional molecules are involved in a significant number of pairings with other “over-connected” objects. Additional file 10: Table S7 lists pairs of significantly connected nodes/objects within the VL-blood dataset that could interact with each other to regulate larger networks. *STAT1,* with 18 interactions was found to be involved in the most number of pairings, followed by *PRKCD, IRF9* and *ISG15*.

### **C**hromosomal “hot spots”: comparison with literature

**Fig. 7:**
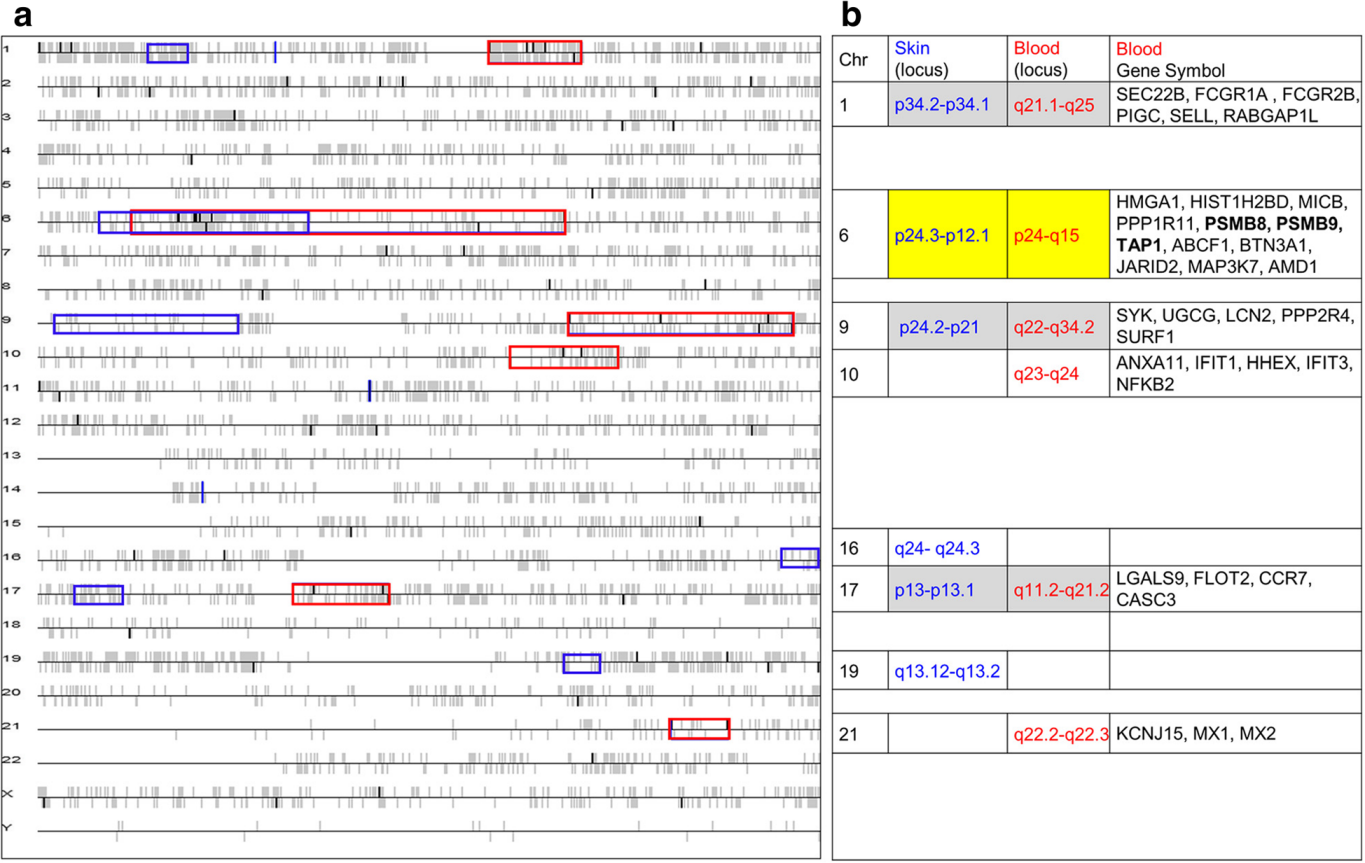
Genome-wide chromosomal distribution of VL-blood and skin DEGs. (**a**) Chromosomal locations of the 99 VL-blood DEGs are colored in bold black vertical bars versus the other genes which are grey. The upright bars above and below the parallel lines represent genes either on the forward or reverse strand. Each horizontal black line corresponds to one chromosome. The VL-blood DEGs were mapped to chromosomes with significant stretches, considered transcriptional “hot spots” marked by 6 *red* boxes. (**b**) A total of 35 dysregulated VL-blood genes are located in the 6 VL-blood “hot spots” with *PSMB8, PSMB9* and *TAP1* (*bold*) on chromosome 6 reported previously as potential VL-associated genetic loci. Subsequently we overlaid the 6 transcriptional “hot spots” from our previous VL-skin analysis (6 *blue* boxes) on the VL-blood chromosomal map. The chromosome # and locus of each transcriptional “hot spot” from VL blood (*red*) and skin (*blue*) is defined. The transcriptionally active “hot spots” on chromosome 6 overlaps between the VL-skin and blood gene expression profiles (*yellow*). Three additional “hot spots” from the two tissue environments map to the same chromosome but do not overlap with each other (*grey*)

Next, we leveraged our VL-blood associated transcriptional profile to map chromosomal areas with an over-representation of probe expression changes as a function of presence of the disease. There is a high expectation of finding disease-associated genetic variations in such transcriptionally active regions, called “hot spots” as demonstrated earlier [56–58]. We identified 6 such regions on chromosomes 1, 6, 9, 10, 17 and 21 harboring a total of 35 down-regulated VL-blood DEGs (Fig. 7a, b). Three “hot spot’ associated genes, *PSMB8* (chr6p21.3), *PSMB9* (chr6p21.3) and *TAP1* (chr6p21.3) overlap with putative VL susceptibility loci [56, 59, 60]. Although HLA-associated genes were not found in our transcriptional dataset, the one transcriptional “hot spot” that is overlapping in both VL-blood and -skin expression profiles, maps to chromosome 6 (chr6p24-q15) and spans the HLA region including, discoidin domain receptor tyrosine kinase 1 (*DDR1*; chr6p21.33) that has been reported as the strongest VL-susceptibility locus [60].

Interestingly, the 35 DEGs mapping within the 6 “hot spots” tether many of the statistically enriched immune/inflammatory canonical pathways and processes such as B-cells-, CD4+ T-cells, *TNF-R2, IL-2, IL-33* and *IL-17* signaling- as well as stress induced apoptosis (Additional file 11: Figure S4 a-d). The canonical *IL-33* signaling pathway enriched in the 35 DEGs included in the VL-blood “hot spots” is coincident with our earlier findings in VL-skin transcriptional profile and linked to inflammation [56]. The *IL-17* signaling pathway has also been increasingly implicated in several AIDs including vitiligo [61]. The remaining 64 VL-skin DEGs identified in our present analysis that did not map within a “hot spot” were enriched in immune related pathways and processes including the IFN α, β and γ, viral process, *IL-4, IL-5,* and *IL-13* mediated inflammatory signaling, as well as negative regulation of cell proliferation. Networks starting with the 64 DEGs outside the “hotspots” tether BPs such as chromatin assembly or disassembly, collagen catabolic processes (data not shown).

Finally, we compared the full set of 99 VL-blood DEGs (35 mapping inside “hot spots”, and 64 outside) to a comprehensive set of 137 VL-associated DEGs and potential gene markers compiled from several GWAS and gene expression studies. Apart from the 3 DEGs (within the “hot spots”) described above that coincided with VL-associated susceptibility genes, *P2RY14* (purinergic receptor P2Y, G-protein coupled, 14) was DIP in the present dataset while being up-regulated in a recent VL-blood microarray study using pooled case and control samples [62]. No other overlaps were found with any other study.

## Discussion

Major gaps remain in our knowledge regarding how genetic and environmental elements alter gene expression at the systemic level to promote or prevent vitiligo. We examined transcriptional profiles from peripheral blood of non-segmental VL cases and healthy controls (including 11 females and 3 males between the ages of 31–70 years, Table 3) using several *in silico* “function based” analyses to better understand underlying regulatory mechanisms in VL-pathogenesis. Our strategy was to integrate genetic, biological and clinical information to identify and characterize molecular elements of potential disease relevance in the blood. The use of unfractionated skin and PBMCs for VL-gene expression analysis offers a global and comprehensive overview of disease-associated transcriptional changes that may be relevant to disease expression and the identification of disease biomarkers.

**Table 3:** Demographic data for study subjects

Sample	Age (Range in yrs.)	Diagnosis	Duration (years)	Current Meds	Previous Treatments	Past Med History
VL1009B	60–69	Vitiligo	10	None	topical and oral steroid	HTN
VL1010B	50–59	Vitiligo	2	None	steroid (1.5 yrs ago)	hypothyroid
VL1016B^a^	70–79	Vitiligo	50	ASA, atenolol, lipitor, detrol LA	Methoxsalen (45–50 yrs. ago), none currently	None
VL1017B^a^	30–39	Vitiligo	10	PABA, vitamin B12, trazodone and other antianxiety med	light treatment (6 yrs. ago), Vit B12 injection	None
VL1018B^a^	30–39	Vitiligo	12	None	None	None
VL1019B^a^	30–39	Vitiligo	26	None	None	Factor V disorder
VL1020B	60–69	Vitiligo	30	None	None	None
VL1021B	60–69	Vitiligo	>20	sunblock	benoquin (depigmentation agent)	rosacea (10yrs ago), osteoarthritis, SCC (10yrs ago)
NL1001B	50–59	No disease	none	none	none	none
NL1004B	30–39	No disease	none	none	none	none
NL1013B	30–39	No disease	none	none	none	none
NL1014B	40–49	No disease	none	none	none	none
NL1020B	50–59	No disease	none	none	none	none
NL1032B	40–49	No disease	none	none	none	none

*Abbreviations*: *VL* vitiligo, *NL* normal, *B* blood, *PABA* para amino benzoic acid, *HTN* hypertension, *SCC* squamous cell carcinoma, *ASA* amino salicylic acid, *LA* long acting. Patients were under no systemic or topical medications for 2 months prior to sampling. ^a^Starred samples are shared between our previous skin and the present blood analyses. Gender and ethnicity details are withheld to protect patient privacy. While the healthy controls were 100% females, the female to male ratio in vitiligo patients is 6.2: 3.8. Ethnicity distribution among healthy controls is 67% African American, 17% Caucasian and 17% Asian and among VL patients 50% Caucasians, 25% Hispanics, 12.5% Asian and 12.5% African

VL-pathogenesis, similar to other heterogeneous autoimmune diseases is multifactorial and polygenic. Unsupervised clustering methods demonstrated a specific “disease-state” based molecular signature in peripheral blood distinguishing VL patients and healthy controls. This signature could potentially be leveraged to identify biomarkers for diagnostic, prognostic and classification purposes. Biological processes (including mostly down-regulated genes) such as immune/defense response and cell activation were found to be enriched in both a subset of VL-blood genes examined from the unbiased hierarchical cluster as well as the differentially expressed gene profile, reinforcing their likely importance as central drivers in disease pathogenesis. Functional annotation of the DEGs using two different analytical platforms (DAVID and MetaCore) illuminated systemically perturbed immune/defense response, protein catabolic processes and antigen processing and presentation enriched in the predominantly down-regulated genes of peripheral blood in VL patients, echoing a recent GWAS study demonstrating an enrichment of similar immuneregulation-related pathways and processes in the 48 confirmed vitiligo associated loci [29].

Broadly, our findings strongly support a down-regulation of both innate and adaptive immune/inflammatory response in VL-blood in the context of an accumulation of regulators in the activation of cytoskeletal remodeling, oxidative stress and apoptosis in VL-skin where the disease is manifest. These findings indicate tissue environment-specific changes in the disease and suggests tightly regulated mechanisms underlying disease-related pathways. The down-regulation of several immune system related pathways in VL blood might represent the susceptibility to the disease in VL patients at the systemic level. Our experiments do not address the temporal order of gene expression alterations, but rather a slice in time. Thus our VL-blood results might be expected to reveal a pattern of gene *dysregulation* that encompasses counter-regulatory molecules/pathways in response to an aberrant immune response.

The overlap of several DEGs within VL-blood profile and disease markers in SLE as well as other AIDs supports existing literature regarding comorbidities in vitiligo patients [10, 12, 52, 54]. Nath et al., (2001) reported evidence of a possible shared genetic effect between vitiligo and SLE, with a probable common genetic determinant on chromosome 17p13 (susceptibility gene *SLEV1*) [53]. In the present analysis of VL-blood, 4 dysregulated genes mapped to the “hot spot” on chromosome 17 in the neighborhood of *SLEV1,* indicating perturbation in the same region.

Both innate and adaptive immune response are induced by pleiotropic cytokines known as IFNs that exhibit antiviral, anti-proliferative and immunomodulatory effects. The migration of transcriptional activator *STAT1* into the nucleus to activate genes involved in cell proliferation and viability can be activated by Type 1 or Type II IFN pathways [63, 64]. Previous investigations have demonstrated that IFN-γ concentrations as well as the IFN-γ: IL-10 ratio in serum plays a major role in VL-induced depigmentation and pathogenesis [47, 65]. Our analysis confirms previous reports supporting [43, 65, 66] the central role for both IFN types in vitiligo. Overall, the present study reinforces the critical role of IFN-γ-chemokine axis for the progression and maintenance of VL, and illuminates a therapeutic potential [43, 67, 68]. Furthermore, the down-regulation of TFs such as *STAT1, STAT6* and *NF-kB* in the VL-blood dataset might be additional indication of the attempt to counter activate apoptotic signals previously observed in lesional skin. Our findings allow us to speculate about the attempt within both the systemic and the target tissue environment to repair and repopulate cells that are defective, dying or dead due to disease.

With regards to “hidden” transcriptional factors, we found several such as *CREB1, MYC, IRF4, IRF1, TP53* in the *VL-blood profile* that echoed a similar TF circuitry found in our previous investigation of *VL-lesional skin* pathology [56], underscoring the importance of the mechanisms relying on the STAT1/IRF9/MYC signaling in the disease. Several transcription factors, including CREB1 and STAT3 are able to modulate the expression and/or transcriptional activity of the microphthalmia transcription factor (*MITF*), a master gene for melanocyte survival [69]. Our VL-skin data previously revealed a down-regulation of genes involved in terminal differentiation of melanocytes. This could be due to the lack of melanocytes in lesional skin as reported in literature [70, 71], or suggest a key role for the breakdown in the signaling pathway involving *MITF* and *CREB1* in melanogenesis. Our results allow us to further consider the participation of the TFs such as *CREB1, MYC, IRF4, STAT1/IRF9, IRF1* and *TP53* in the regulation of viral processes, IFN signaling pathway, innate immune response, immune effector processes, response to cytokine stimulus, defense response, and cellular response to Type I and II IFNs. We had previously noted In VL-skin that melanocytes, immune cells and keratinocytes, among other cell types experience proliferative signals accompanied by a pronounced up-regulation of nucleotide and protein metabolic processes.

The enriched BCR pathway (including DEGs: *FCGR2B, NFKB2, MAP3K7, SYK* and *PTPN6*) that is part of the ‘adaptive’ cellular response [72] plays a critical role in the development, survival, and activation of B lymphocytes. This pathway is composed of membrane immunoglobulin molecules which bind antigens causing receptor aggregations and act through several transduction molecules such as *SYK* and *MAP3K7* that influence TFs such as NF-kB in the nucleus, permitting several distinct outcomes, including proliferation, differentiation, apoptosis, survival and tolerance of B cells. The down-regulation of all VL-blood genes that are represented in the pathway might contribute to the switchboard functioning of the BCR pathway, enabling it to selectively turn on a specific signaling pathways by keeping others quiescent [73, 74].

Studies have demonstrated the usefulness of using gene expression data to successfully prioritize candidate genes for disease-associated single nucleotide polymorphisms in genome-wide studies [75]. Our analysis mapped 6 “hot spots” as transcriptionally active sites with *TAP1* (transporter 1, ATP-binding cassette, subfamily B member), *PSMB8* and *PSMB9* (proteasome subunit, beta type, 8 and 9) coinciding with previously reported VL susceptibility loci [76]. These three genes are linked to macromolecular catabolic processes associated with proteasomes which normally protect against the development of T-cell mediated AIDs [77]. Proteasomes are responsible for degrading short-lived cytoplasmic proteins into peptides [78]. Among its 28 subunits, the 20*S* proteasome includes two subunits known as *PSMB8* (*LMP7*) and *PSMB9* (*LMP2*). Anomalies in TAP1 and *PSMB* proteins have been reported to be associated with vitiligo along with several other AIDs such as Sjogren’s syndrome, type 1 diabetes, juvenile rheumatoid arthritis, celiac disease, and multiple sclerosis [79, 80]. *TAP1* functions by providing candidate peptides to MHC-I molecules within the peptide-loading complex and by transferring antigenic peptides from the cytoplasm into the endoplasmic reticulum [81]. The significance of all three proteasome-related molecules being down-regulated in blood, might indicate that coordinated events of healthy antigen processing and presentation have gone awry in VL patients. Interruption of the self-antigen presentation pathway on MHC-I has been shown to be a possible pathway for self-tolerance and autoreactivity against a variety of target organs [82, 83]. Similar to our published results in VL-skin, the “hot spot” region mapping to chromosome 6 in VL-blood also covers the same HLA region coinciding with the strongest genetic associations of VL [59, 60]. The influence of the HLA region is also likely connected to its well-established role in antigen presentation and T cell activation [84].

Working towards personalized alternate therapy choices in VL, we broadened our search for key molecules related to the disease that can potentially be targeted by drugs. We focused on five DEGs (*STAT1, PRKCD*, *PTPN6, MYC* and *FGFR2)* based on the following criteria: 1) differential expression in VL-skin or blood; 2) inclusion in disease-related pathways; 3) interactome analysis highlighting “over-connectivity” in functional interactions; 4) network analysis demonstrating all 5 targets as interactive hubs, and 5) paired functional interacting units. Crucial regulatory roles are indicated for the two transcriptional factors (*STAT1* and *MYC*), the phosphatase (*PTPN6*) and kinase (*PRKCD*) in VL pathogenesis. Although *FGFR2* is not one of the “over-connected” DEGs, the localization of the disease-related growth receptor in the membrane with an extracellular domain capable of being recognized by specific ligands and drugs, makes it an attractive [85] molecule to investigate as a potential target. Interestingly, among the five molecules delineated by the present study (by a combination of transcriptome and bioinformatics analyses), *STAT1* is presently being investigated as a promising target in the treatment of VL [68, 86]. The lack of *FGFR2* expression results in a melanocyte-related disease such as piebaldism. Overall, we suggest the following five molecules to be prioritized as targets for future potential VL-therapy:

1. ***Signal Transducer and Activator of Transcription 1*** *,* (*STAT1*) (FC = -2.6; VL-blood) is a 91kDa member of the STAT family that reacts to cytokines and growth factors. It is phosphorylated by the receptor associated kinases and translocates to the cell nucleus where it acts as a transcription activator involved in apoptosis. While responding to *IFN-γ, STAT1* is phosphorylated, and regulated by activation of *PRKCD* downstream of the activation of the phosphatidylinositol 3-kinase (PI3K) signaling pathway. This can result in either pro- or anti-apoptotic outcomes depending on the cellular context and other interacting pathways [87, 88]. *STAT1* has also been shown to act as a driver in cancers, modulating downstream *MYC* expression, which in turn promotes the capacity for proliferation, migration, and invasion of cells [89]. *STAT1* activation that is essential for IFN-γ signaling has been targeted by simvastatin in mouse models (FDA-approved medication for lowering cholesterol levels) and is currently being investigated [68].
2. ***Protein Kinase C, delta*** (*PRKCD*) (FC = -1.7; VL-blood) is a member of the family of serine- and threonine-specific protein kinases that can be activated by calcium. Human and mouse studies demonstrate that this kinase is involved in B cell signaling, regulation of growth, apoptosis, and differentiation of a variety of cell types [90], as well as the IFN-γ signaling described above [88].
3. ***Protein Tyrosine Phosphatase, Non-Receptor Type 6*** (*PTPN6)* (FC = -1.9; VL-blood) encodes for a member of the protein tyrosine phosphatase (PTP) family of signaling molecules that regulate a range of cellular processes such as cell growth, differentiation and mitotic cycle, while itself being regulated by the cytokine IFN-α. PTPN6 was shown to be a negative regulator of EMT transition which is involved in loss of cell-cell adhesion and increase in cell motility and reorganization in skin [91]. Cytokine-based therapies have the potential to provide novel treatments for cancer, infectious disease, and AIDS.
4. We have described ***v-myc avian myelocytomatosis viral oncogene homolog*** (*c-Myc*/*MYC*) (FC = 1.5; VL-skin) as an “over-connected” TF in our previous VL-skin report [56]. It is involved in cell growth, apoptosis and metabolism. It functions as a transcription factor regulating specific target genes. The *MYC* proto-oncogene has been found to be stimulated in various animal and human tumors. It is important in both development as well as cell proliferation.
5. ***Fibroblast growth factor receptor 2*** (*FGFR2*) (FC = 1.9 VL-skin) is a highly conserved protein. It is part of the FGFR family whose members differ from each other in ligand affinities and tissue distribution. The maturation and continued existence of migrating melanoblasts is closely associated with simultaneous *FGFR2* expression and a lack or dysfunction of the receptor results in maladies such as piebaldism (related to melanocyte development), among others [85]. A loss of *FGFR2* function is also shown to contribute to melanoma [92]. *FGFR2* has an extracellular portion that interacts with fibroblast growth factors, setting in motion a cascade of downstream signals ultimately influencing mitogenesis and differentiation.

## Conclusions and future directions

The present study represents a genome-wide transcriptome analysis of blood, from predominantly female non-segmental vitiligo patients and healthy controls, examined in the context of our previous VL-skin report. Using several *in silico* bioinformatics-based analyses, we identify five novel molecules (*STAT1, PRKCD, PTPN6, MYC* and *FGFR2*) that have the potential to be targeted by drugs for future therapy. Additionally, we reveal molecular regulators affecting apoptosis, cytoskeletal remodeling, oxidative stress and metabolism in the skin and immune response in the blood that are suggested to contribute to the autoimmune reaction against melanocytes in the skin, similar to a recent report [29]. Future work will involve a larger cohort of different sub-phenotypes of VL patients, both male and female, accompanied with cell sorting of purified cell populations with the aim of assigning DEGs to specific candidate cell types. Longitudinal analyses of VL lesions that are newly developing, flaring or undergoing re-pigmentation may help to illuminate specific transcriptional changes within a temporal framework of disease development and progression. On-going integrated analyses of transcriptional regulation in tissue-specific and circulatory environments has the potential to clarify details of molecular interplay that tethers the autoimmune response underlying disease pathogenesis in vitiligo. Future research into available drugs that can target the five vital disease-linked molecules proposed in this report holds the promise of expanding efficacious treatment in vitiligo.

## Methods

A diagnosis of non-segmental VL (referred to as vitiligo or VL in this paper) based on established clinical criteria was the basis for recruitment to the study at the Dermatology outpatient clinic of New York Presbyterian Hospital-Cornell University. Ethical guidelines were followed by obtaining signed consent forms for punch biopsies and blood draw from patients and healthy controls (IRB # 0998-398). PBMCs were isolated from blood of VL patients and healthy control individuals that were all off therapy at the time of sampling. All specimens were snap frozen in liquid nitrogen immediately. The procedures for blood and tissue handling, PBMC extractions, total RNA preparation, cDNA synthesis have been described before [93–95]. Demographic details with age, duration of disease, ethnicity and treatment history for each subject that were chosen for subsequent analysis are presented in Table 3.

### Microarray method

The Affymetrix GeneChip Expression Analysis Technical Manual (Affymetrix, Santa Clara, CA, http://www.affymetrix.com) was followed for experimental procedures for microarray assays as described earlier [56, 94, 95]. Labeled cRNA was hybridized for 16 h at 45°C to microarrays (Affymetrix HG-U95Av1_v2). The chips were then washed, stained and scanned according to manufacturer’s protocol (Affymetrix Inc., Santa Clara, CA) on the Affymetrix Fluidics Station 750, and scanned by the Affymetrix GeneChip Scanner 3000.

### Microarray data analysis

Gene expression values from lesional/non-lesional skin (n-16) and blood samples (*n* = 24) from VL patients and healthy controls were imported into Partek Genomics Suite v6.6 (Partek, St Louis, MO) as CEL files. Raw data preprocessing details have been described earlier [56]. The data was examined using quality control criteria in Partek software. Ten normal blood samples were discarded from the final differential expression analysis due to a failure to pass quality control check. Expression data from peripheral blood of VL patients (*n* = 8, 5 females and 3 males) and healthy controls (*n* = 6, all females) were finally used for establishing DEGs with “disease-state” as the greatest source of variation across all 12,625 probeset IDs. Average expression levels were distributed similarly across all samples.

### Unsupervised hierarchical clustering

The informative probe sets with the most variation across arrays were selected for ‘unbiased’ hierarchical cluster analysis using a coefficient of variation filter greater than 0.12 across all blood arrays. We found 1346 most variably expressed probeset IDs from peripheral blood samples of VL cases and controls. A two way cluster analysis was performed on these probe sets based on Euclidean distance and centroid linkage (1-r metric) for samples and average linkage for probeset IDs. Subsequently, we examined these 1346 probeset IDs to find a set of 319 non-redundant transcripts that were down-regulated in VL patients and functionally annotated them via DAVID. Principal components analysis was performed on all 12,625 probeset IDs (transformed and normalized) to look for key variables in the dataset and observe batch effects in dataset. We superimposed sample information using color coding after assessing the sample separations by the unbiased clustering methods.

### Differentially expressed genes (DEGs)

We defined DEGs between VL patients and healthy controls using similar methods as described before [56]. While controlling the *p*-value at <0.05, we used a FC cut off ≥ ±1.4 to generate a list of 105 DEGs. A trimmed list of 99 unique DEGs (upon removing redundancies, non-annotated probeset IDs and pseudogenes) was generated and used for all subsequent analyses. Array data is also internally validated by examining multiple probeset IDs for the same gene. When data from all such probes in the experiment are identified as correlating in an array experiment, there is higher probability of the finding being real [96]. For e.g. annotation for Probeset IDs: 263_g_at (FC-1.57), 262_at (FC = --1.50) and 36685_at (FC = -1.50) were all *AMD1* (adenosylmethionine decarboxylase 1). We finally retained the representative probeset ID associated with the highest +/-fold change linked with the lowest *p*-value among the replicates to generate the list of ﻿*non-redundant* DEG﻿s﻿. Using both unbiased and supervised methods to reveal corroborating results serves to further bolster the disease-related findings in our report.

Tools in DAVID [97] (https://david.ncifcrf.gov/) as well MetaCore™ v6.21 (Thomson Reuters, St Joseph, MI) (http://www.genego.com) were used for ontology enrichment analyses [98] [99]. Methods were the same as described earlier [56]. We were able to generate and analyze several disease-associated canonical pathway maps in MetaCore database followed by similar investigations in the Gene Ontology (GO) and KEGG databases.

### Chromosomal mapping and interactome/network analyses

We leveraged the VL-blood gene expression data to delineate regions of chromosomal enrichment (“hot spots”) that contain more dysregulated genes than can be expected by chance [58, 100]. *P-value ≤ 0.05* was calculated for all stretches of chromosomes that contained ≥ 3 DEGs (from the 99 VL-blood DEGs used for this analysis) and these significant stretches (“hot spots”) are outlined in boxes (Fig. 7a). The DEGs in the VL-blood expression profile were analyzed in the context of all the >12,625 genes on the array for their chromosomal location enrichment using DNA-Chip Analyzer (dChip) (www.dchip.org) using instructions as detailed in https://sites.google.com/site/dchipsoft/high-level-analysis/map-genes-to-chromosome. Duplicate probe sets were masked for gene mapping using the “genome” tool as described earlier [56, 93, 95]. We subsequently overlaid the VL-skin chromosomal map on the VL-blood map to investigate overlapping “hot spots” between the two environments. In order to find key molecular elements underlying disease pathology in VL patients we used an *in silico* global protein interaction network analysis of the VL-blood transcriptional dataset [101]. The relative connectivity of a gene or protein reflects its functional consequence to VL [102]. It is calculated by the number of interactions between an experimental gene with the other genes on the experimental list normalized to the number of interactions it has with all genes in the human database (Metabase). One hundred and sixty VL-associated DEGs, potential biomarkers and susceptibility loci were discovered from VL-associated genes found by microarray and GWAS (http://www.genome.gov) [26, 29, 32, 33, 43, 56, 62, 70, 76, 79, 103–135] as well as the metabase and were compared to our DEGs list to search for overlaps.

## Additional files

Additional file 1: Table S1. Potential disease relevant GO biological processes associated with down-regulated genes discovered by unsupervised hierarchical clustering. (PDF 87 kb)
Additional file 2: Table S2. Differentially expressed genes in VL peripheral blood transcriptional profile. (PDF 320 kb)
Additional file 3: Table S3. Ontology enrichment analysis of VL DEGs (peripheral blood). (PDF 118 kb)
Additional file 4: Figure S1. Enriched canonical pathway related to immune response. (PDF 830 kb)
Additional file 5: Figure S2. Profile trellis: VL-blood associated DEGs. (PDF 770 kb)
Additional file 6: Table S4. Enrichment by Protein Function. (PDF 97 kb)
Additional file 7: Table S5. Interactome topology and over-connected genes. (PDF 106 kb)
Additional file 8: Figure S3. Network analysis of sixteen “over-connected” genes. (PDF 1934 kb)
Additional file 9: Table S6. Transcriptional regulation network list from VL-blood associated experimental dataset. (PDF 196 kb)
Additional file 10: Table S7. Significant paired nodes within over-connected functional genes. (PDF 192 kb)
Additional file 11: Figure S4. Ontology enrichment analysis of 35 VL-blood associated DEGs that mapped to six chromosomal “hot spots”. (PDF 901 kb)

## Abbreviations

AID: Autoimmune disorders
BCR: B-Cell antigen receptor
BP: Biological processes
CD8 + T: Cytotoxic T lymphocytes
DAVID: Database for annotation, visualization and integrated discovery
DEGs: Differentially expressed genes
DIP: Down-regulated in patients
FC: Fold change
GO: Gene ontology
GWAS: Genome-wide association studies
HLA: Human leukocyte antigen
IFN: Interferon
IL-2: Interleukin-2
ISG-15: Interferon-stimulated gene 15
NF-kB: Nuclear factor kappa-light-chain-enhancer of activated B cells
PBMC: Peripheral blood mononuclear cell
PCA: Principal components analysis
SLE: Systemic lupus erythematosus
TF/s: Transcriptional factor/s
UIP: Upregulated in patients
VL: Vitiligo
