# Supplementary material for: Vitiligo blood transcriptomics provides new insights into disease mechanisms and identifies potential novel therapeutic targets
Source: BMC Genomics. 2017 Jan 28;18:109. doi: 10.1186/s12864-017-3510-3 (PMC5273810; doi:10.1186/s12864-017-3510-3)
Supplement: Additional file 1: Table S1. — Potential disease relevant GO biological processes associated with down-regulated genes discovered by unsupervised hierarchical clustering. (PDF 87 kb) [file 12864_2017_3510_MOESM1_ESM.pdf]

GO #	Biological Processes	Count	Fold Enrichment	p-value
* 0006955	immune response	61	4.4	1.27E-22
* 0001775	cell activation	25	4.3	3.9E-09
* 0009615	response to virus	16	7.2	4.75E-09
0045321	leukocyte activation	22	4.5	2E-08
0002521	leukocyte differentiation	15	5.7	3.96E-07
* 0002684	positive regulation of immune system process	19	3.9	1.6E-06
0051249	regulation of lymphocyte activation	15	5.0	1.75E-06
0002520	immune system development	20	3.6	3.35E-06
0048534	hemopoietic or lymphoid organ development	19	3.6	5.59E-06
* 0006952	defense response	31	2.5	6.97E-06
0050865	regulation of cell activation	15	4.2	1.25E-05
0007242	intracellular signaling cascade	48	1.9	2.33E-05
0006954	inflammatory response	20	3.0	3.36E-05
0002757	immune response-activating signal transduction	8	7.6	7.77E-05
0002252	immune effector process	12	4.4	8.47E-05
0030097	hemopoiesis	16	3.3	9.05E-05
0002429	immune response-activating cell surface receptor signaling pathway	7	8.9	0.000119
0002764	immune response-regulating signal transduction	8	7.1	0.000126
* 0050863	regulation of T cell activation	11	4.6	0.000127
0002768	immune response-regulating cell surface receptor signaling pathway	7	8.2	0.000182

Additional File 1: Table S1. Potential disease relevant GO biological processes associated with down-regulated genes discovered by unsupervised hierarchical clustering. Unbiased analysis was able to use the most variably expressed genes (see Materials and Methods) from the peripheral blood of 8 VL patients and 6 healthy control individuals and cluster them into a distinct signature based upon "disease state". From the total of 1346 variable genes included in this analysis, a group of 319 were subsequently revealed to be down-regulated in VL patients. Functional annotation of the down-regulated genes by DAVID revealed enrichment in gene ontology (GO) biological processes that have previously been implicated in the clinical setting of VL. **Count**, # of DEGS in the list involved in the specific biological process. Starred* biological processes are shared with biological processes enriched in the 99 differentially expressed genes (DEGs) obtained subsequently from comparing the two groups of samples.

Running title: VL-blood transcriptomics identifies potential novel therapeutic targets. **Dey-Rao and Sinha 2016**
