# Supplementary material for: Vitiligo blood transcriptomics provides new insights into disease mechanisms and identifies potential novel therapeutic targets
Source: BMC Genomics. 2017 Jan 28;18:109. doi: 10.1186/s12864-017-3510-3 (PMC5273810; doi:10.1186/s12864-017-3510-3)
Supplement: Additional file 2: Table S2. — Differentially expressed genes in VL peripheral blood transcriptional profile. (PDF 320 kb) [file 12864_2017_3510_MOESM2_ESM.pdf]

Entrez Gene	Gene Symbol	Chrom Location	Gene Title	p-value	Fold-Change (Vitiligo vs. Control)
23	ABCF1	chr6p21.33	ATP-binding cassette, sub-family F (GCN20), member 1	0.0361	-1.4
262	AMD1	chr6q21	adenosylmethionine decarboxylase 1	0.0192	-1.6
23253	ANKRD12	chr18p11.22	ankyrin repeat domain 12	0.0236	1.4
311	ANXA11	chr10q23	annexin A11	0.0485	-1.4
11119	BTN3A1	chr6p22.1	butyrophilin, subfamily 3, member A1	0.0453	-1.5
22794	CASC3	chr17q21.1	cancer susceptibility candidate 3	0.0269	-1.5
1236	CCR7	chr17q12-q21.2	chemokine (C-C motif) receptor 7	0.0472	-2.1
5218	CDK14	chr7q21-q22	cyclin-dependent kinase 14	0.0197	-1.5
8760	CDS2	chr20p13	CDP-diacylglycerol synthase (phosphatidate cytidylyltransferase) 2	0.0314	-1.5
27243	CHMP2A	chr19q	charged multivesicular body protein 2A	0.0409	-1.9
91782	CHMP7	chr8p21.3	charged multivesicular body protein 7	0.0237	-1.5
11316	COPE	chr19p13.11	coatomer protein complex, subunit epsilon	0.0142	-1.5
10404	CPQ	chr8q22.2	carboxypeptidase Q	0.0221	-1.6
1545	CYP1B1	chr2p22.2	cytochrome P450, family 1, subfamily B, polypeptide 1	0.0069	1.4
1577	CYP3A5	chr7q21.1	cytochrome P450, family 3, subfamily A, polypeptide 5	0.0315	1.5
9416	DDX23	chr12q13.12	DEAD (Asp-Glu-Ala-Asp) box polypeptide 23	0.0233	-1.5
1796	DOK1	chr2p13	docking protein 1, 62kDa (downstream of tyrosine kinase 1)	0.0428	-1.5

5610	EIF2AK2	chr2p22-p21	eukaryotic translation initiation factor 2-alpha kinase 2	0.0107	-1.7
2000	ELF4	chrXq26	E74-like factor 4 (ets domain transcription factor)	0.0363	-1.7
2201	FBN2	chr5q23-q31	fibrillin 2	0.0346	1.5
2209	FCGR1A	chr1q21.2-q21.3	Fc fragment of IgG, high affinity Ia, receptor (CD64) /// Fc fragment of IgG, high affi	0.0067	-2.7
2213	FCGR2B	chr1q23	Fc fragment of IgG, low affinity IIb, receptor (CD32)	0.0429	-1.4
2319	FLOT2	chr17q11-q12	flotillin 2	0.0345	-1.6
3087	HHEX	chr10q23.33	hematopoietically expressed homeobox	0.0469	-1.5
3017	HIST1H2BD	chr6p21.3	histone cluster 1, H2bd	0.0128	-1.9
3159	HMGA1	chr6p21	high mobility group AT-hook 1	0.0340	-1.4
10561	IFI44	chr1p31.1	interferon-induced protein 44	0.0221	-2.2
10964	IFI44L	chr1p31.1	interferon-induced protein 44-like	0.0284	-3.6
3434	IFIT1	chr10q23.31	interferon-induced protein with tetratricopeptide repeats 1	0.0169	-4.0
3437	IFIT3	chr10q24	interferon-induced protein with tetratricopeptide repeats 3	0.0134	-3.5
10581	IFITM2	chr11p15.5	interferon induced transmembrane protein 2	0.0342	-2.7
10379	IRF9	chr14q11.2	interferon regulatory factor 9	0.0049	-1.7
9636	ISG15	chr1p36.33	ISG15 ubiquitin-like modifier	0.0173	-4.2
3669	ISG20	chr15q26	interferon stimulated exonuclease gene 20kDa	0.0225	-2.2

3720	JARID2	chr6p24-p23	jumonji, AT rich interactive domain 2	0.0296	-1.6
7994	KAT6A	chr8p11	K(lysine) acetyltransferase 6A	0.0461	-1.8
3772	KCNJ15	chr21q22.2	potassium inwardly-rectifying channel, subfamily J, member 15	0.0119	-1.9
22992	KDM2A	chr11q13.2	lysine (K)-specific demethylase 2A	0.0482	-1.6
9903	KLHL21	chr1p36.31	kelch-like family member 21	0.0268	-1.4
3934	LCN2	chr9q34	lipocalin 2	0.0244	-1.4
3965	LGALS9	chr17q11.2	lectin, galactoside-binding, soluble, 9	0.0125	-1.9
57149	LYRM1	chr16p11.2	LYR motif containing 1	0.0280	-1.5
6885	MAP3K7	chr6q15	mitogen-activated protein kinase kinase kinase 7	0.0334	-1.5
23383	MAU2	chr19p13.11	MAU2 chromatid cohesion factor homolog (C. elegans)	0.0338	-1.5
79143	MBOAT7	chr19q13.4	membrane bound O-acyltransferase domain containing 7	0.0382	-1.8
9968	MED12	chrXq13	mediator complex subunit 12	0.0218	-1.5
4236	MFAP1	chr15q15-q21	microfibrillar-associated protein 1	0.0380	-1.6
4277	MICB	chr6p21.3	MHC class I polypeptide-related sequence B	0.0085	-3.3
4489	MT1A	chr16q13	metallothionein 1A	0.0001	-1.7
4599	MX1	chr21q22.3	myxovirus (influenza virus) resistance 1, interferon-inducible protein p78 (mouse)	0.0099	-3.5
4600	MX2	chr21q22.3	myxovirus (influenza virus) resistance 2 (mouse)	0.0193	-2.2
653361	NCF1	chr7q11.23	neutrophil cytosolic factor 1	0.0436	-2.7
4689	NCF4	chr22q13.1	neutrophil cytosolic factor 4, 40kDa	0.0389	-1.8

8440	NCK2	chr2q12	NCK adaptor protein 2	0.0065	-1.8
4738	NEDD8	chr14q12	neural precursor cell expressed, developmentally down-regulated 8	0.0173	-1.5
4791	NFKB2	chr10q24	nuclear factor of kappa light polypeptide gene enhancer in B-cells 2 (p49/p100)	0.0206	-1.5
25936	NSL1	chr1q41	NSL1, MIND kinetochore complex component, homolog (S. cerevisiae)	0.0249	-1.6
8638	OASL	chr12q24.2	2'-5'-oligoadenylate synthetase-like	0.0334	-2.7
9934	P2RY14	chr3q24-q25.1	purinergic receptor P2Y, G-protein coupled, 14	0.0105	-1.6
5279	PIGC	chr1q23-q25	uncharacterized LOC100505991 /// phosphatidylinositol glycan anchor biosynthesis, class	0.0256	-1.6
11040	PIM2	chrXp11.23	pim-2 oncogene	0.0103	-1.5
5359	PLSCR1	chr3q23	phospholipid scramblase 1	0.0149	-2.1
6992	PPP1R11	chr6p21.3	protein phosphatase 1, regulatory (inhibitor) subunit 11	0.0402	-1.5
5524	PPP2R4	chr9q34	protein phosphatase 2A activator, regulatory subunit 4	0.0367	-1.5
5580	PRKCD	chr3p21.31	protein kinase C, delta	0.0318	-1.7
23550	PSD4	chr2q13	pleckstrin and Sec7 domain containing 4	0.0069	-1.6
5696	PSMB8	chr6p21.3	proteasome (prosome, macropain) subunit, beta type, 8	0.0210	-1.6
5698	PSMB9	chr6p21.3	proteasome (prosome, macropain) subunit, beta type, 9	0.0421	-1.7
5777	PTPN6	chr12p13	protein tyrosine phosphatase, non-receptor type 6	0.0302	-1.9

23369	PUM2	chr2p22-p21	pumilio homolog 2 (Drosophila)	0.0406	-1.6
9910	RABGAP1L	chr1q24	RAB GTPase activating protein 1-like	0.0403	-1.7
23108	RAP1GAP2	chr17p13.3	RAP1 GTPase activating protein 2	0.0113	-1.5
6210	RPS15A	chr16p	ribosomal protein S15a	0.0368	-1.8
91543	RSAD2	chr2p25.2	radical S-adenosyl methionine domain containing 2	0.0487	-2.1
9554	SEC22B	chr1q21.1	vesicle-trafficking protein SEC22b-like /// SEC22 vesicle trafficking protein homolog B	0.0050	-1.5
6402	SELL	chr1q23-q25	selectin L	0.0492	-1.7
710	SERPING1	chr11q12.1	serpin peptidase inhibitor, clade G (C1 inhibitor), member 1	0.0084	-4.0
6672	SP100	chr2q37.1	SP100 nuclear antigen	0.0427	-1.5
6689	SPIB	chr19q13.3-q13.4	Spi-B transcription factor (Spi-1/PU.1 related)	0.0013	-1.6
6772	STAT1	chr2q32.2	signal transducer and activator of transcription 1, 91kDa	0.0289	-2.6
6778	STAT6	chr12q13	signal transducer and activator of transcription 6, interleukin-4 induced	0.0441	-1.5
6801	STRN	chr2p22.2	striatin, calmodulin binding protein	0.0323	-1.5
6827	SUPT4H1	chr17q21-q23	suppressor of Ty 4 homolog 1 (S. cerevisiae)	0.0306	-1.5
6834	SURF1	chr9q34.2	surfeit 1	0.0428	-1.5
6850	SYK	chr9q22	spleen tyrosine kinase	0.0323	-1.7
6890	TAP1	chr6p21.3	transporter 1, ATP-binding cassette, sub-family B (MDR/TAP)	0.0097	-1.6
23435	TARDBP	chr1p36.22	TAR DNA binding protein	0.0453	-1.7

23102	TBC1D2B	chr15q24.3-q25.1	TBC1 domain family, member 2B	0.0137	-1.5
10953	TOMM34	chr20q12-q13.1	translocase of outer mitochondrial membrane 34	0.0371	-1.6
9881	TRANK1	chr3p22.2	tetratricopeptide repeat and ankyrin repeat containing 1	0.0371	-2.0
6737	TRIM21	chr11p15.5	tripartite motif containing 21	0.0058	-1.6
9246	UBE2L6	chr11q12	ubiquitin-conjugating enzyme E2L 6	0.0322	-1.7
23304	UBR2	chr6p21.1	ubiquitin protein ligase E3 component n-recognin 2	0.0461	-1.4
23352	UBR4	chr1p36.13	ubiquitin protein ligase E3 component n-recognin 4	0.0210	-1.6
7357	UGCG	chr9q31	UDP-glucose ceramide glucosyltransferase	0.0014	-1.9
147179	WIPF2	chr17q21.2	WAS/WASL interacting protein family, member 2	0.0450	-1.6
9189	ZBED1	chrXp22.33; Yp11	zinc finger, BED-type containing 1	0.0140	-1.9
22882	ZHX2	chr8q24.13	zinc fingers and homeoboxes 2	0.0118	-1.6
7572	ZNF24	chr18q12	zinc finger protein 24	0.0481	-1.5

Additional File 2: Table S2. Differentially expressed genes in VL peripheral blood transcriptional profile. Microarray analysis comparing peripheral blood dataset from 8 VL patients 6 healthy controls generated 99 non-redundant differentially expressed genes (DEGs). FC=Fold Change; a “positive” fold change indicates an upregulation and a "negative” fold change indicates a down-regulation in Vitiligo patients vs.healthy control individuals. VL= Vitiligo. Chrom location= chromosomal location.

Running title: VL-blood transcriptomics identifies potential novel therapeutic targets. **Dey-Rao and Sinha 2016**
