# Supplementary material for: Vitiligo blood transcriptomics provides new insights into disease mechanisms and identifies potential novel therapeutic targets
Source: BMC Genomics. 2017 Jan 28;18:109. doi: 10.1186/s12864-017-3510-3 (PMC5273810; doi:10.1186/s12864-017-3510-3)
Supplement: Additional file 3: Table S3. — Ontology enrichment analysis of VL DEGs (peripheral blood). (PDF 118 kb) [file 12864_2017_3510_MOESM3_ESM.pdf]

a

KEGG Pathway Term	Homo sapiens Hsa #	Fold Enrichment	p-value	DEGs
Fc gamma R-mediated phagocytosis	04666	6.8	0.0051	*FCGR1C, FCGR2B, NCF1, FCGR1A, NCF1C, PRKCD, SYK*
Systemic lupus erythematosus	05322	5.2	0.0369	*HIST1H2BD, FCGR1C, FCGR2B, FCGR1A, TRIM21*

b

Biological Process Term	GO Accession #	Fold Enrichment	p-value	DEGs
response to virus	0009615	17.3	7.00E-11	*LCN2, IRF9, PLSCR1, ISG15, RSAD2, RPS15A, IFI44, MX1, EIF2AK2, STAT1, MX2, ISG20*
immune response	0006955	4.6	4.00E-08	*MICB, SP100, IFITM2, NCF1, NCF4, NCF1C, RSAD2, IFI44L, SERPING1, NFKB2, PRKCD, PSMB8, PSMB9, OASL, CCR7, FCGR2B, FCGR1C, FCGR1A, P2RY14, TAP1, ANXA11, FCGR1B*
regulation of immune effector process	0002697	7.8	4.00E-03	*MAP3K7, STAT6, MICB, SERPING1, SYK*
cellular macromolecule catabolic process	0044265	2.6	6.00E-03	*ISG15, KDM2A, UBR4, ZHX2, UBE2L6, KLHL21, NEDD8, UBR2, CASC3, PSMB8, ISG20, PSMB9*
cell activation	0001775	3.8	9.00E-03	*PLSCR1, NCK2, MICB, ELF4, NFKB2, PRKCD, SYK*
regulation of cellular protein metabolic process	0032268	3	1.00E-02	*NCK2, PPP2R4, SERPING1, CASC3, PUM2, PRKCD, PSMB8, PSMB9, SYK*
defense response	0006952	2.6	1.00E-02	*ABCF1, CCR7, SP100, FCGR1C, NCF1, FCGR1A, TAP1, NCF1C, RSAD2,*

				SERPING1, MX1, MX2
negative regulation of macromolecule metabolic process	0010605	2.4	2.00E-02	*HHEX, SP100, JARID2, ZHX2, ZNF24, PPP2R4, SERPING1, SUPT4H1, PRKCD, PSMB8, PSMB9*
positive regulation of immune system process	0002684	4	2.00E-02	*MAP3K7, STAT6, NCK2, MICB, SERPING1, SYK*
positive regulation of lymphocyte activation	0051251	6.5	2.00E-02	*MAP3K7, STAT6, NCK2, SYK*
immune response-activating cell surface receptor signaling pathway	0002429	12.1	2.00E-02	*MAP3K7, MICB, SYK*
negative regulation of response to stimulus	0048585	6.3	3.00E-02	*STAT6, MICB, SERPING1, PRKCD*
negative regulation of cellular protein metabolic process	0032269	4.4	3.00E-02	*PPP2R4, SERPING1, PRKCD, PSMB8, PSMB9*
modification-dependent protein catabolic process	0019941	2.5	3.00E-02	*ISG15, KDM2A, UBR4, UBE2L6, KLHL21, NEDD8, UBR2, PSMB8, PSMB9*
immune response-regulating cell surface receptor signaling pathway	0002768	11.2	3.00E-02	*MAP3K7, MICB, SYK*
positive regulation of leukocyte activation	0002696	5.9	3.00E-02	*MAP3K7, STAT6, NCK2, SYK*
ISG15-protein conjugation	0032020	62.9	3.00E-02	*ISG15, UBE2L6*
positive regulation of cell activation	0050867	5.7	3.00E-02	*MAP3K7, STAT6, NCK2, SYK*
proteolysis involved in cellular protein catabolic process	0051603	2.4	3.00E-02	*ISG15, KDM2A, UBR4, UBE2L6, KLHL21, NEDD8, UBR2, PSMB8, PSMB9*
lymphocyte activation	0046649	4	4.00E-02	*NCK2, MICB, ELF4, PRKCD, SYK*
regulation of transcription from RNA polymerase II promoter	0006357	2.2	4.00E-02	*STAT6, HHEX, SP100, ELF4, JARID2, ZHX2, MED12, SPIB, NEDD8, SUPT4H1*
positive regulation of macromolecule metabolic process	0010604	2	4.00E-02	*STAT6, HHEX, SP100, ELF4, MED12, PPP2R4, SUPT4H1, HMGA1, PSMB8, PSMB9, SYK*

immune response-activating signal transduction	0002757	9.1	4.00E-02	*MAP3K7, MICB, SYK*
T cell activation	0042110	5	4.00E-02	*NCK2, MICB, ELF4, SYK*

Additional File 3: Table S3. Ontology enrichment analysis of VL DEGs (peripheral blood). Microarray analysis from peripheral blood of 8 VL patients and 6 healthy control individuals generated 99 non-redundant differentially expressed genes (DEGs). Ontology enrichment analysis by DAVID reveals several enriched dysregulated and disease-related **(a)** KEGG pathways and **(b)** Gene Ontology (GO) biological processes.

Running title: VL-blood transcriptomics identifies potential novel therapeutic targets. **Dey-Rao and Sinha 2016**
