# Supplementary material for: Vitiligo blood transcriptomics provides new insights into disease mechanisms and identifies potential novel therapeutic targets
Source: BMC Genomics. 2017 Jan 28;18:109. doi: 10.1186/s12864-017-3510-3 (PMC5273810; doi:10.1186/s12864-017-3510-3)
Supplement: Additional file 4: Figure S1. — Enriched canonical pathway related to immune response. (PDF 830 kb) [file 12864_2017_3510_MOESM4_ESM.pdf]

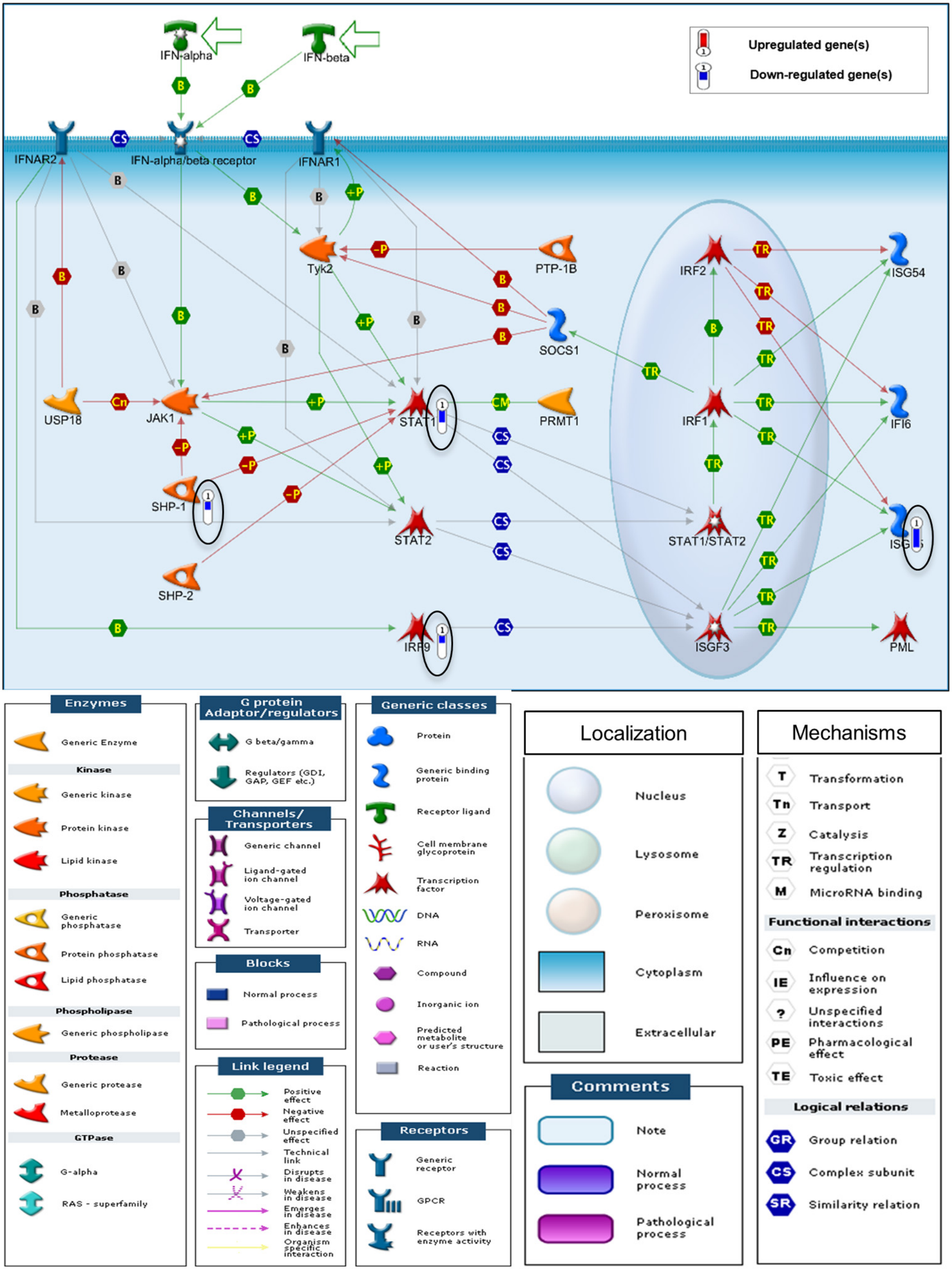

Additional File 4: Figure S1.

Enriched canonical pathway related to immune response. IFN alpha/beta signaling pathway is the most highly enriched canonical pathway in the 99 VL-blood DEGs. (See Figure 2) The genes from the VL-blood expression profile are down-regulated and circled in black. SHP-1 is synonymous with PTPN6. Experimental data is visualized on the maps as blue (for downregulation) and red (upregulation) histograms. The height of the histogram corresponds to the relative expression value for a particular gene/protein. Types of objects in the pathway are clarified in the legend.

Running title: VL-blood transcriptomics identifies potential novel therapeutic targets. **Dey-Rao and Sinha 2016**
