# Supplementary material for: Vitiligo blood transcriptomics provides new insights into disease mechanisms and identifies potential novel therapeutic targets
Source: BMC Genomics. 2017 Jan 28;18:109. doi: 10.1186/s12864-017-3510-3 (PMC5273810; doi:10.1186/s12864-017-3510-3)
Supplement: Additional file 5: Figure S2. — Profile trellis: VL-blood associated DEGs. (PDF 770 kb) [file 12864_2017_3510_MOESM5_ESM.pdf]

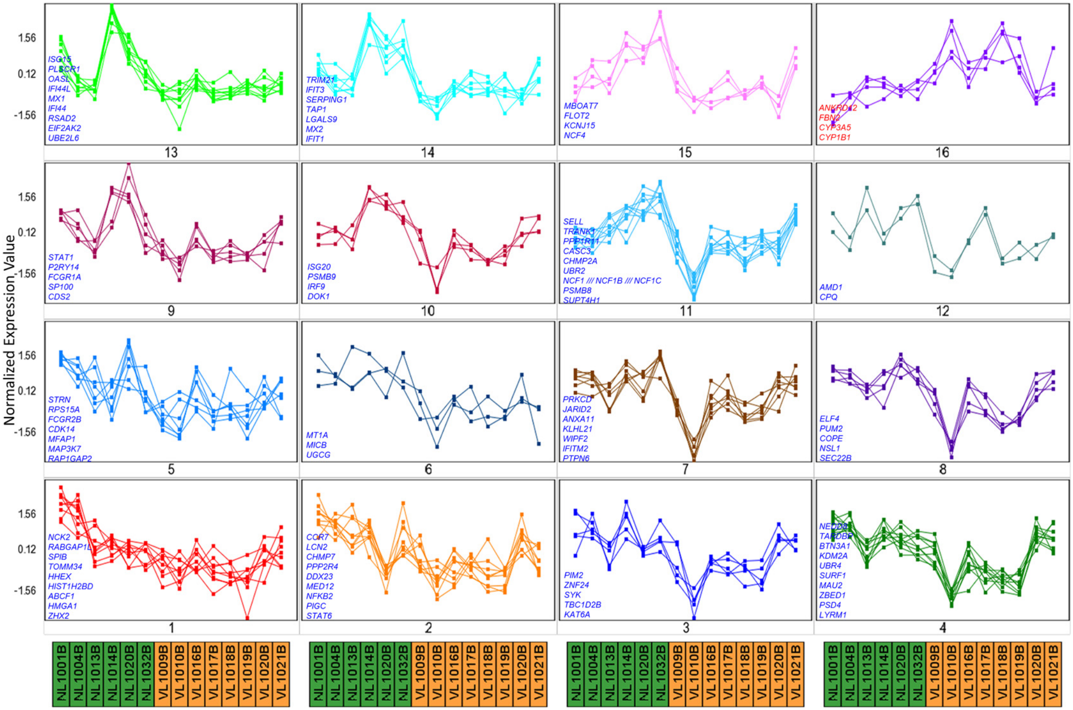

Additional File 5: Figure S2.

Profile trellis: VL-blood associated DEGs.

To study gene expression patterns of 99 DEGs in all samples, we visualized GCRMA processed, normalized expression values (standardized-shift rows to mean of zero and scaled to standard deviation of one) of each dysregulated genes associated with VL-blood transcriptional profile. We created self-organizing maps where clustering is performed on rows and the number of clusters is equal to the imposed map height and width of 4 X 4 with 20,000 training iterations. Fifteen groups showed the expected net down-regulation of 95 genes (blue) and 1 group with 4 genes showed an overall upregulation (red) in vitiligo patients (**orange**) as opposed to healthy controls (**green**).

Running title: VL-blood transcriptomics identifies potential novel therapeutic targets. **Dey-Rao and Sinha 2016**
