# Supplementary material for: Vitiligo blood transcriptomics provides new insights into disease mechanisms and identifies potential novel therapeutic targets
Source: BMC Genomics. 2017 Jan 28;18:109. doi: 10.1186/s12864-017-3510-3 (PMC5273810; doi:10.1186/s12864-017-3510-3)
Supplement: Additional file 6: Table S4. — Enrichment by Protein Function. (PDF 97 kb) [file 12864_2017_3510_MOESM6_ESM.pdf]

**Protein class**	**Actual**	**n**	**R**	**N**	**E**	**Ratio**	**p-value**	**z-score**	**In data set %**	**In protein function %**	**Protein function in database %**
**Transcription factors**	**14**	**102**	**976**	**26494**	**3.758**	**3.726**	**2.26E-05**	**5.394**	**13.73**	**1.43**	**3.68**
Phosphatases	2	102	228	26494	0.8778	2.278	0.2191	1.205	1.96	0.88	0.86
Kinases	5	102	657	26494	2.529	1.977	0.1099	1.576	4.90	0.76	2.48
Proteases	4	102	579	26494	2.229	1.794	0.1844	1.202	3.92	0.69	2.19
**Enzymes**	**18**	**102**	**2774**	**26494**	**10.68**	**1.685**	**0.01859**	**2.372**	**17.65**	**0.65**	**10.47**
Receptors	5	102	1615	26494	6.218	0.8042	0.4051	-0.5049	4.90	0.31	6.10
Ligands	1	102	514	26494	1.979	0.5053	0.4086	-0.704	0.98	0.19	1.94
Other	53	102	19204	26494	73.93	0.7169	7.93E-06	-4.65	51.96	0.28	72.48

Additional File 6: Table S4. Enrichment by Protein Function. VL- peripheral blood DEGs list was activated and corresponding network objects were mapped in MetaCore. We then conducted an enrichment analysis by protein function that scores and ranks the most relevant protein functions related to the activated dataset. Transcription factors (14) and Enzymes (18) (**in bold**) are the only two significant functional categories.

Explanation of each column: Protein class: a broadly defined protein function; Actual; number of network objects from the activated dataset(s) for a given protein class; n: total number of network objects in the activated dataset(s); R: total number of network objects of a given protein class in the complete database or background list; N: total number of network objects in the complete database or background list; E: # of objects that would be expected to occur by chance, mean value for hypergeometric distribution (n*R/N); Ratio: connectivity ratio (Actual/Expected); z-score: {(Actual-Expected)/sqrt (variance)}; p-value: probability to have the given value of Actual or higher (or lower for negative z-score); In data set %: fraction of network objects with a selected function in the activated dataset; In protein function: fraction of network with a selected function in the activated dataset among network objects with this function in the complete database or background list; protein function in database: fraction of network objects with a selected function in the complete database or background list.

Running title: VL-blood transcriptomics identifies potential novel therapeutic targets. **Dey-Rao and Sinha 2016**
