# Supplementary material for: Vitiligo blood transcriptomics provides new insights into disease mechanisms and identifies potential novel therapeutic targets
Source: BMC Genomics. 2017 Jan 28;18:109. doi: 10.1186/s12864-017-3510-3 (PMC5273810; doi:10.1186/s12864-017-3510-3)
Supplement: Additional file 7: Table S5. — Interactome topology and over-connected genes. (PDF 106 kb) [file 12864_2017_3510_MOESM7_ESM.pdf]

a

	Complete database	Experiment(s)	Experiment(s) only
**Degree**	22.41	64.93	1.333
**Degree-in**	11.21	25.95	0.6667
**Degree-out**	11.21	38.98	0.6667
**Clustering coefficient**	0.07351	0.09235	0.07107

b

#	IDs in active data set	Object name	Actual	n	R	N	E	Ratio	z-score	p-value
1		IRF1	16	102	199	26494	0.7661	20.88	17.5	6E-17
**2**	***STAT1***	**STAT1**	**17**	**102**	**332**	**26494**	**1.2782**	**13.30**	**14.0**	**1.13E-14**
3		IRF4	15	102	429	26494	1.6516	9.08	10.5	1.05E-10
4		STAT2	8	102	68	26494	0.2618	30.56	15.2	2.23E-10
**5**	***IRF9***	**IRF9**	**5**	**102**	**20**	**26494**	**0.0770**	**64.94**	**17.8**	**1.13E-08**
6		PU.1	12	102	376	26494	1.4476	8.29	8.9	2.41E-08
7		IRF2	6	102	73	26494	0.2810	21.35	10.8	3.88E-07
8		IRF8	9	102	250	26494	0.9625	9.35	8.2	5.48E-07
9		MYC (c-Myc)	26	102	2431	26494	9.3592	2.78	5.7	1.17E-06
10		c-Rel (NF-kB subunit)	8	102	208	26494	0.8008	9.99	8.1	1.47E-06

Additional File 7: Table S5. Interactome topology and over-connected genes. We annotated the VL-peripheral blood profile and subjected it to interactome analysis: **(a) Interactome topology:** The experimental VL-blood dataset shows connectivity (coming in and going out) to objects in the metabase with a higher clustering coefficient (0.09235) than either, only among objects within the entire metabase (0.07351) or VL- blood profile (0.07107). **(b) Interactions of transcription factors (TFs).** The top 10 TFs show significant over-connections to other objects in the experimental VL- blood dataset as well as the larger metabase that exceed the expected number, and are accompanied by low p-values and high z-scores. The two VL-blood TFs (**bold**) with significant over-connections are ***IRF9 and STAT1***. Although, the remaining eight TFs are not found in the experimental VL-blood dataset, they are significantly over-connected with objects within the dataset (actual) as well as in the metabase (R) and sorted by p-value.

Explanation of each column and row:

A) Degree: average number of interactions per node; Degree in: average number of ingoing interactions per node; Degree-out: average number of outgoing interactions per node; Clustering coefficient: average clustering coefficient for node; Complete database: calculated for the complete database or background list; Experiment(s): calculated for the complete database; average is calculated for the experimental set; Experiment(s) only: calculated for the experimental set (zero values in this column means that there is no interactions between objects from experimental set.

B) IDs in active data sets: gene symbol associated with the VL-blood transcriptional profile; Object Name: network objects (transcription factors) in metabase; Actual: Actual number of network objects in the activated dataset which interact with the chosen object; n: number of network objects in the activated dataset; R: number of network objects in the complete database or background list which interact with the chosen object; N: total number of objects in the background list; E: Expected mean of hypergeometric distribution (n*R/N); Ratio: connectivity ratio (Actual/Expected); z-score: (Actual-Expected)/ (standard deviation); p-value: probability to have the given value of Actual or higher by chance under null hypothesis of over-connectivity.

Running title: VL-blood transcriptomics identifies potential novel therapeutic targets. **Dey-Rao and Sinha 2016**
