# Supplementary material for: Vitiligo blood transcriptomics provides new insights into disease mechanisms and identifies potential novel therapeutic targets
Source: BMC Genomics. 2017 Jan 28;18:109. doi: 10.1186/s12864-017-3510-3 (PMC5273810; doi:10.1186/s12864-017-3510-3)
Supplement: Additional file 8: Figure S3. — Network analysis of sixteen “over-connected” genes. (PDF 1934 kb) [file 12864_2017_3510_MOESM8_ESM.pdf]

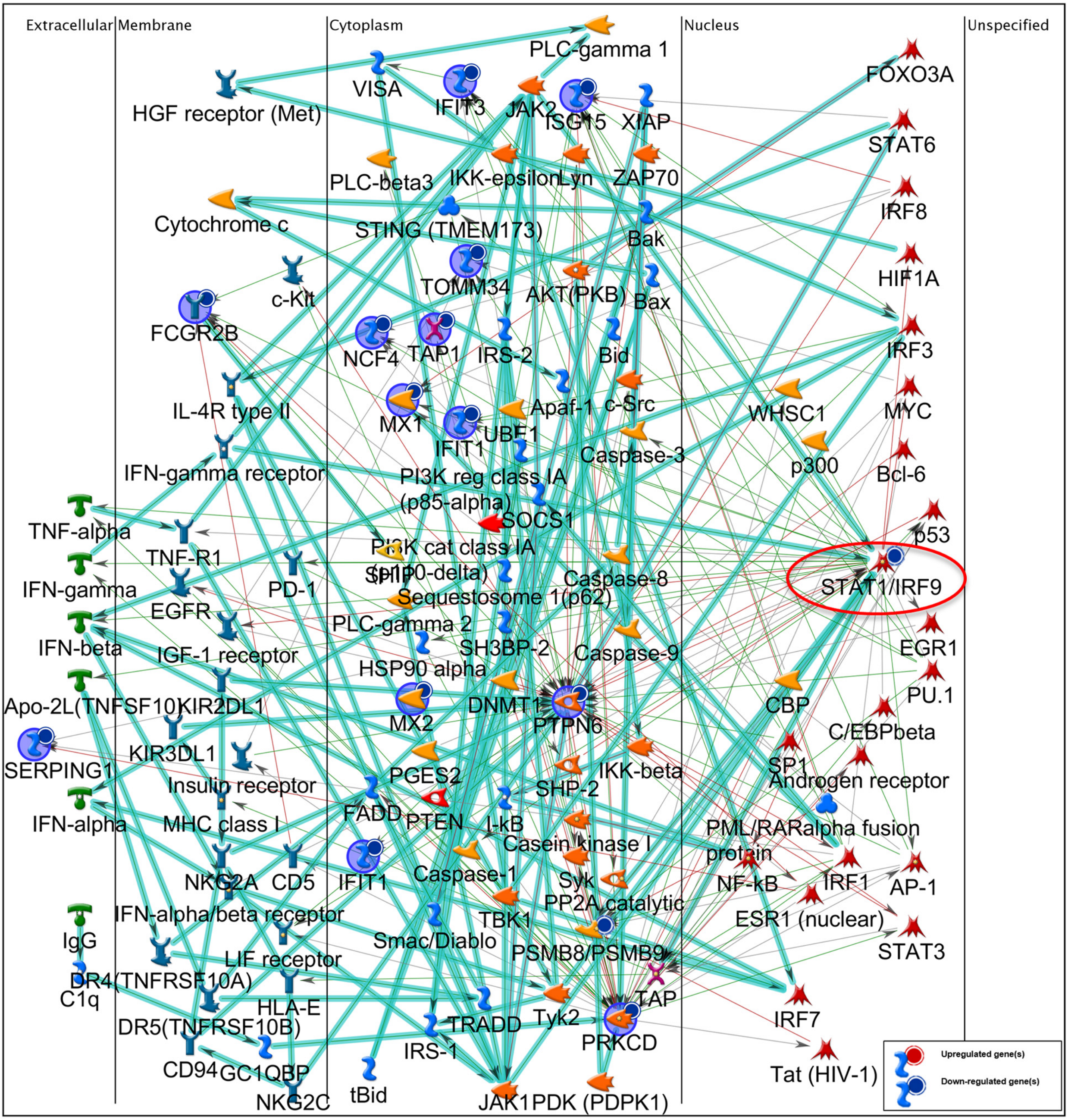

Additional File 8 Figure S3. Network analysis of sixteen "over-connected" genes. Sixteen genes were found to be "over-connected" by function in the VL-blood transcriptional profile (13 down-regulated genes form seed nodes, encircled in **blue**). Of the "over-connected molecules, STAT1/IRF9 and PSMB8/PSMB9 mapped to protein complexes and as such, while not embedded inside blue circles, represent seed nodes as well. Networks were generated using the shortest path algorithm and expanded by 2 objects. STAT1/IRF9 (**red** circle) form a central hub of incoming and outgoing (direction of arrowheads), positive green lines) and negative (**red** lines) interactions including portions of several canonical pathways (represented by thick **cyan** lines). Genes (encoding proteins) have been ordered according to their cellular localization.. For legend see Figure 4.

Running title: VL-blood transcriptomics identifies potential novel therapeutic targets. **Dey-Rao and Sinha 2016**
