# Supplementary material for: Vitiligo blood transcriptomics provides new insights into disease mechanisms and identifies potential novel therapeutic targets
Source: BMC Genomics. 2017 Jan 28;18:109. doi: 10.1186/s12864-017-3510-3 (PMC5273810; doi:10.1186/s12864-017-3510-3)
Supplement: Additional file 9: Table S6. — Transcriptional regulation network list from VL-blood associated experimental dataset. (PDF 196 kb) [file 12864_2017_3510_MOESM9_ESM.pdf]

Transcriptional regulation network list from VL-blood gene expression profile						
#	Network	GO processes	Total nodes	Seed nodes	p-Value	z-Score
1	CREB1	regulation of viral process (18.6%; 2.749e-08), type I interferon signaling pathway (14.0%; 2.931e-08), innate immune response (32.6%; 3.050e-08), cellular response to type I interferon (14.0%; 3.131e-08), response to type I interferon (14.0%; 3.341e-08)	45	44	4.24E-133	197.26
2	MYC (c-Myc)	innate immune response (46.2%; 2.929e-13), immune effector process (35.9%; 3.741e-12), response to cytokine (41.0%; 5.190e-12), defense response (51.3%; 1.954e-11), immune response (48.7%; 2.022e-11)	40	39	1.85E-117	185.45
3	IRF4	innate immune response (63.3%; 2.291e-17), type I interferon signaling pathway (33.3%; 3.768e-17), cellular response to type I interferon (33.3%; 4.224e-17), immune effector process (53.3%; 4.334e-17), response to type I interferon (33.3%; 4.729e-17)	31	30	1.17E-89	162.03
4	STAT1	immune effector process (63.0%; 8.347e-20), immune response (77.8%; 1.638e-18), cellular response to cytokine stimulus (63.0%; 2.440e-18), response to cytokine (66.7%; 2.443e-18), cellular response to type I interferon (37.0%; 1.199e-17)	28	28	5.33E-85	159.13
5	IRF1	defense response (82.1%; 7.320e-20), innate immune response (71.4%; 8.107e-20), type I interferon signaling pathway (39.3%; 1.046e-19), cellular response to type I interferon (39.3%; 1.187e-19), response to type I interferon (39.3%; 1.346e-19)	29	28	1.55E-83	156.36
6	SP1	immune system process (75.0%; 2.173e-13), immune effector process (46.4%; 4.134e-13), response to organic substance (78.6%; 2.288e-12), response to cytokine (50.0%; 3.967e-12), cellular response to cytokine stimulus (46.4%; 5.174e-12)	29	28	1.55E-83	156.36

7	TP53 (p53)	immune effector process (52.2%; 5.602e-13), immune system process (73.9%; 6.683e-11), response to cytokine (52.2%; 7.362e-11), immune response (60.9%; 1.796e-10), innate immune response (52.2%; 4.621e-10)	24	23	2.5E-68	141.18
8	PU.1	immune system process (85.7%; 1.764e-13), cytokine-mediated signaling pathway (52.4%; 4.045e-13), immune response (71.4%; 1.196e-12), immune effector process (52.4%; 5.035e-12), response to cytokine (57.1%; 1.744e-11)	22	21	2.78E-62	134.63
9	c-Fos	response to cytokine (66.7%; 1.557e-14), immune response (71.4%; 1.196e-12), cellular response to cytokine stimulus (57.1%; 1.367e-12), cellular response to chemical stimulus (85.7%; 1.802e-12), innate immune response (61.9%; 4.327e-12)	22	21	2.78E-62	134.63
10	RelA (p65 NF-kB subunit)	immune response (81.0%; 9.739e-16), innate immune response (71.4%; 3.648e-15), defense response (81.0%; 8.787e-15), response to cytokine (66.7%; 1.557e-14), immune effector process (57.1%; 1.288e-13)	22	21	2.78E-62	134.63
11	STAT2	immune effector process (75.0%; 2.007e-19), innate immune response (80.0%; 1.928e-17), defense response (90.0%; 3.030e-17), cellular response to type I interferon (45.0%; 6.190e-17), response to type I interferon (45.0%; 6.850e-17)	21	20	2.88E-59	131.24
12	c-Jun	response to cytokine (70.0%; 5.421e-15), cellular response to chemical stimulus (90.0%; 3.010e-13), cellular response to organic substance (85.0%; 3.686e-13), immune response (75.0%; 3.691e-13), cellular response to cytokine stimulus (60.0%; 6.064e-13)	21	20	2.88E-59	131.24
13	IRF8	immune response (90.0%; 2.874e-18), innate immune response (80.0%; 1.928e-17), cytokine-mediated signaling pathway (65.0%; 5.261e-17), immune effector process (65.0%; 1.071e-15), defense response (85.0%; 1.831e-15)	21	20	2.88E-59	131.24

14	HIF1A	response to cytokine (63.2%; 3.258e-12), response to other organism (63.2%; 3.122e-11), response to external biotic stimulus (63.2%; 3.185e-11), immune effector process (52.6%; 4.536e-11), response to biotic stimulus (63.2%; 4.832e-11)	20	19	2.95E-56	127.75
15	IRF7	innate immune response (84.2%; 4.059e-18), immune effector process (73.7%; 5.061e-18), cytokine-mediated signaling pathway (68.4%; 1.882e-17), response to cytokine (78.9%; 2.689e-17), immune response (89.5%; 3.253e-17)	20	19	2.95E-56	127.75

Additional File 9: Table S6. Transcriptional regulation network list from VL-blood associated experimental dataset.
The VL-blood gene expression profile (DEGs list) was analyzed by the transcriptional regulation network algorithm in MetaCore which lists the top 15 out of 30 TFs associated with networks and gene ontology (GO) biological processes that are ranked by p-value and z-score.

Explanation of columns: **Network:** Transcription factor; **GO processes:** denotes the various gene ontology biological processes associated with the network. **Total nodes:** total number of objects in the network; **seed nodes**: number of objects from the experimental dataset; **p-value:** represents the probability of intersection between the experimental dataset and the prebuilt content in MetaCore occurring purely by chance; **z-score:** calculates the level of saturation of the networks taking into account the size of the database, the number of objects in the subnetwork and the number of objects in the experimental signature used to construct the network. The higher the z-score the more saturated a subnetwork is with experimental data.

Running title: VL-blood transcriptomics identifies potential novel therapeutic targets.
Dey-Rao and Sinha 2016
