# Supplementary material for: Vitiligo blood transcriptomics provides new insights into disease mechanisms and identifies potential novel therapeutic targets
Source: BMC Genomics. 2017 Jan 28;18:109. doi: 10.1186/s12864-017-3510-3 (PMC5273810; doi:10.1186/s12864-017-3510-3)
Supplement: Additional file 10: Table S7. — Significant paired nodes within over-connected functional genes. (PDF 192 kb) [file 12864_2017_3510_MOESM10_ESM.pdf]

#	IDs in active data set	Gene Title	IDs of corresponding network object from Network	Gene Title
1	EIF2AK2	eukaryotic translation initiation factor 2-alpha kinase 2	IRF9	interferon regulatory factor 9
			ISG15	ISG15 ubiquitin-like modifier
			**STAT1**	signal transducer and activator of transcription 1, 91kDa
2	IFIT1	interferon-induced protein with tetratricopeptide repeats 1	IFIT1	interferon-induced protein with tetratricopeptide repeats 1
			IFIT3	interferon-induced protein with tetratricopeptide repeats 3
			IFIT3	
			IRF9	interferon regulatory factor 9
			IRF9	
			ISG15	ISG15 ubiquitin-like modifier
			**STAT1**	ISG15 ubiquitin-like modifier
			**STAT1**	
3	IFIT3	interferon-induced protein with tetratricopeptide repeats 3	IRF9	interferon regulatory factor 9
			ISG15	ISG15 ubiquitin-like modifier
			**STAT1**	signal transducer and activator of transcription 1, 91kDa
4	IRF9	interferon regulatory factor 9	ISG15	ISG15 ubiquitin-like modifier
			STAT6	signal transducer and activator of transcription 6, interleukin-4 induced
5	*MX1; MX2*	*myxovirus (influenza virus) resistance 1, interferon-inducible protein p78 (mouse): myxovirus (influenza virus) resistance 2 (mouse)*	*ISG15*	*ISG15 ubiquitin-like modifier*
			SP100	SP100 nuclear antigen
			**STAT1**	signal transducer and activator of transcription 1, 91kDa
6	NCF4	neutrophil cytosolic factor 4, 40kDa	PRKCD	protein kinase C, delta

			**STAT1**	signal transducer and activator of transcription 1, 91kDa
7	P2RY14	purinergic receptor P2Y, G-protein coupled, 14	PRKCD	protein kinase C, delta
8	PLSCR1	phospholipid scramblase 1	PRKCD	
			**STAT1**	signal transducer and activator of transcription 1, 91kDa
9	PSMB9	PSMB9	PSMB8	proteasome (prosome, macropain) subunit, beta type, 8
10	PTPN6	protein tyrosine phosphatase, non-receptor type 6	FCGR2B	Fc fragment of IgG, low affinity IIb, receptor (CD32)
			PRKCD	protein kinase C, delta
			**STAT1**	signal transducer and activator of transcription 1, 91kDa
			STAT6	signal transducer and activator of transcription 6, interleukin-4 induced
			SYK	spleen tyrosine kinase
11	SEC22B	vesicle-trafficking protein SEC22b-like /// SEC22 vesicle trafficking protein homolog B	PRKCD	protein kinase C, delta
			**STAT1**	signal transducer and activator of transcription 1, 91kDa
12	**STAT1**	signal transducer and activator of transcription 1, 91kDa	IRF9	interferon regulatory factor 9
			ISG15	ISG15 ubiquitin-like modifier
			PRKCD	protein kinase C, delta
			PSMB9	proteasome (prosome, macropain) subunit, beta type, 9
			SERPING1	serpin peptidase inhibitor, clade G (C1 inhibitor), member 1
			SP100	SP100 nuclear antigen
			STAT6	signal transducer and activator of transcription 6, interleukin-4 induced
			SYK	spleen tyrosine kinase

			TAP1	transporter 1, ATP-binding cassette, sub-family B (MDR/TAP)
13	STAT6	signal transducer and activator of transcription 6, interleukin-4 induced	ISG15	ISG15 ubiquitin-like modifier
14	SYK	spleen tyrosine kinase	FCGR2B	Fc fragment of IgG, low affinity IIb, receptor (CD32)
15	TAP1	transporter 1, ATP-binding cassette, sub-family B (MDR/TAP)	PRKCD	protein kinase C, delta
			PSMB8	proteasome (prosome, macropain) subunit, beta type, 8
			PSMB9	proteasome (prosome, macropain) subunit, beta type, 9
16	TOMM34	translocase of outer mitochondrial membrane 34	SYK	spleen tyrosine kinase
17	UBE2L6	ubiquitin-conjugating enzyme E2L 6	ISG15	ISG15 ubiquitin-like modifier

Additional File 10: Table S7. Significant paired nodes within over-connected functional genes: We activate the annotated VL-blood dataset that includes 99 DEGs and focus on the "over-connected" genes (encoding proteins) by function (see Table 2). We identify objects that pass the FDR threshold of 0.05 in significance, to reveal pairs of key nodes within our dataset that interact with one another to regulate a larger network. The transcription factor STAT1 (**bold**) is the highest interacting partner involved in such interactions followed by PRKCD, IRF9 and ISG15.

Explanation of columns:

IDs in active data set: over-connected proteins (Gene IDs) in the activated VL-blood profile found by Interactome analysis.

Interacting IDs in active data set: over-connected proteins (Gene IDs) found by interactome analysis that act as a pair with IDs in active data set.

Running title: VL-blood transcriptomics identifies potential novel therapeutic targets. **Dey-Rao and Sinha 2016**
