# Supplementary material for: Vitiligo blood transcriptomics provides new insights into disease mechanisms and identifies potential novel therapeutic targets
Source: BMC Genomics. 2017 Jan 28;18:109. doi: 10.1186/s12864-017-3510-3 (PMC5273810; doi:10.1186/s12864-017-3510-3)
Supplement: Additional file 11: Figure S4. — Ontology enrichment analysis of 35 VL-blood associated DEGs that mapped to six chromosomal “hot spots”. (PDF 901 kb) [file 12864_2017_3510_MOESM11_ESM.pdf]

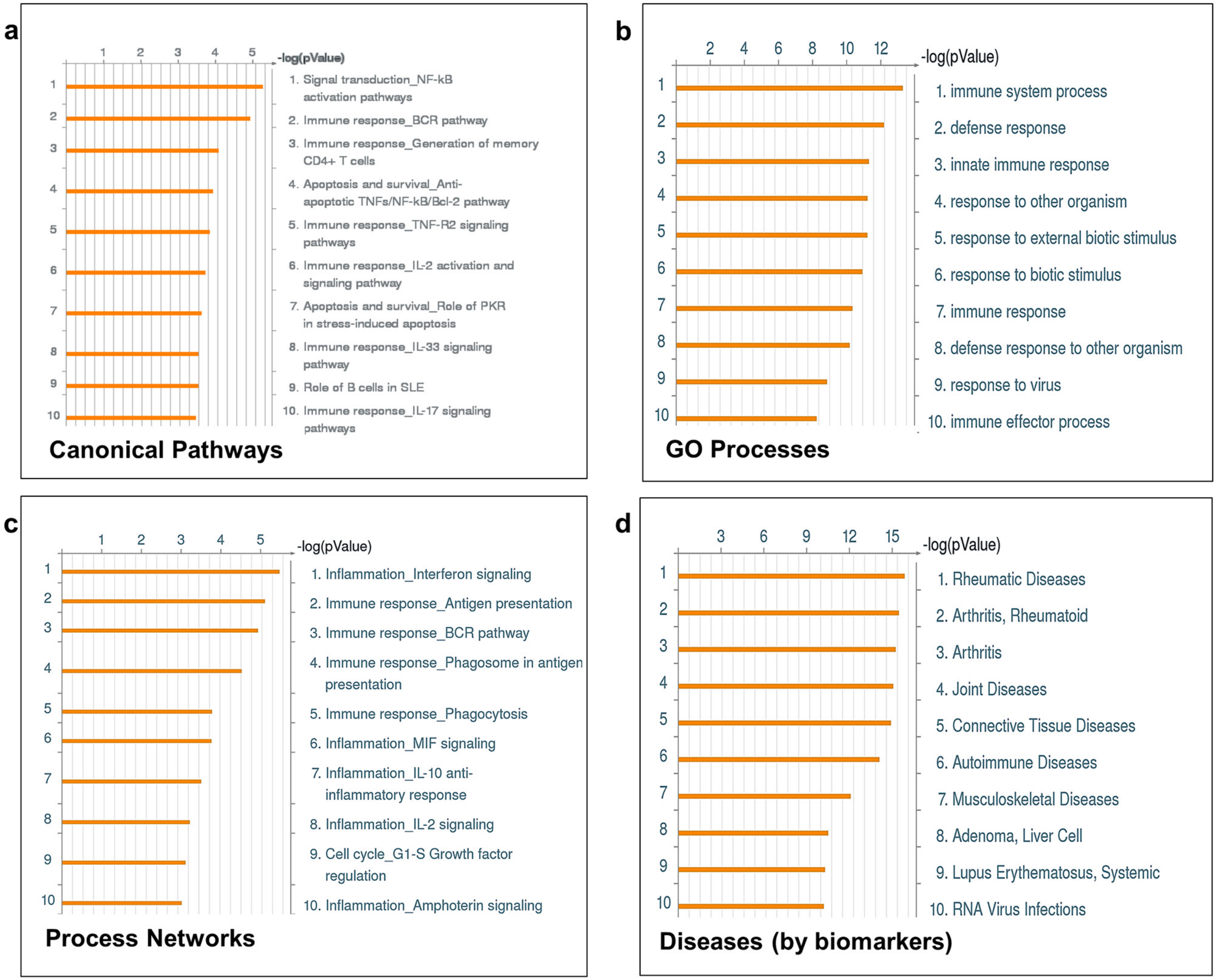

Additional File 11: Figure S4. Ontology enrichment analysis of 35 VL-blood associated DEGs that mapped to six chromosomal "hot spots".

Chromosomal distribution of the genome-wide VL-blood expression dataset with 99 DEGs revealed six transcriptional "hot spots" where the DEGs mapped in significant stretches. Ontology enrichment analysis was performed on the 35 DEGs included within the 6 "hot spots", demonstrating an over-representation in the following categories: (**a**) canonical pathways (**b**) GO processes (**c**) process networks and (**d**) diseases by biomarkers. Top ten enrichments are sorted and ranked by p-value shown on a logarithmic scale. A lower p-value indicates higher statistical significance.

Running title: VL-blood transcriptomics identifies potential novel therapeutic targets. **Dey-Rao and Sinha 2016**
